# NREP, transcriptionally upregulated by HIF-1α, aggravates breast cancer cell growth and metastasis by promoting glycolysis

**DOI:** 10.1038/s41420-024-01951-2

**Published:** 2024-05-02

**Authors:** Yuxia Ruan, Jianghua Qiao, Jiabin Wang, Zhenzhen Liu

**Affiliations:** Department of Breast Disease, Henan Breast Cancer Center, The Affiliated Cancer Hospital of Zhengzhou University & Henan Cancer Hospital, Zhengzhou, 450008 China

**Keywords:** Cancer metabolism, Prognostic markers

## Abstract

Breast cancer (BC) poses a great threat to women’s health. Neuronal regeneration related protein (NREP) is a multifunctional protein that is involved in embryonic development, regeneration, and human disease. However, the biological function of NREP in tumors is rarely reported and its role in BC remains unknown. Bioinformatics analysis showed that NREP is highly expressed and closely correlated with poor survival in BC patients. Under hypoxic conditions, NREP was upregulated in BC cells, and this promotion was reversed by hypoxia-inducible factor HIF-1α suppression. Luciferase reporter system and chromatin immunoprecipitation assays confirmed that HIF-1α directly binds to the promoter of NREP to increase the transcriptional activity of NREP. NREP suppression inhibited cell proliferation, arrested the cell cycle at the G1/S phase, and promoted apoptosis and caspase-3 activity in BC cells. Suppression of NREP decreased the tube formation ability of HUVECs. In addition, NREP downregulation showed an inhibition effect on cell migration, invasion, and EMT of BC cells. In NREP overexpressed cells, all these changes were reversed. In vivo, animal experiments also confirmed that NREP promotes BC tumor growth and metastasis. In addition, NREP promoted cellular glycolysis and enhanced the levels of glucose consumption, ATP, lactate production, and glucose transporters expression in NREP-overexpressed BC cells. In summary, our results demonstrated that NREP could be transcriptional activated by HIF-1α, which may aggravate BC tumor growth and metastasis by promoting cellular glycolysis. This result suggested that NREP may play an essential part in BC progression.

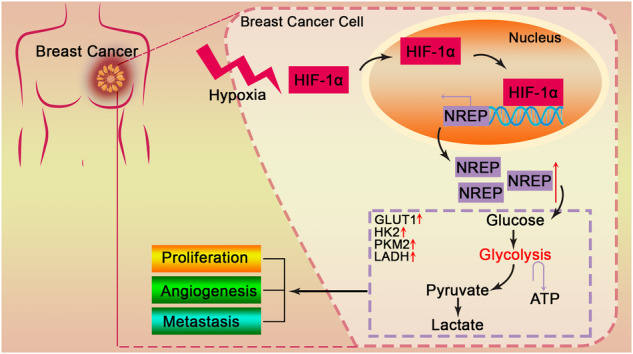

## Introduction

Breast cancer (BC) is a complex disease that displays both inter- and intra-tumoral heterogeneity [1] and is the most frequently diagnosed cancer and the leading cause of cancer-related death among women worldwide [2]. It also has a complicated pathogenesis, including hormone disorders, genetics, environment, etc. Surgery, chemotherapy, hormone therapy, and radiotherapy are common treatments for BC clinical patients [3]. However, metastasis and recurrence remain the main causes of poor survival in BC patients, even though significant advances have been made in the treatment of primary BC [4–6]. Therefore, it is of great significance to explore the key factors during tumor progression, which may alleviate the clinical outcome of BC.

Neuronal regeneration related protein (NREP) is a conserved RNA-binding intracellular protein containing only 68 amino acids [7]. NREP was first identified by Studler and colleagues [8] and is relatively conserved in many species [9], expressed in the brain, smooth muscles, regenerated tissue, and malignant glioblastomas [10]. NREP plays an essential role in promoting wound closure [11]. Reported studies showed that NREP promotes the proliferation and migration of fibroblasts [12–14], and the migration of hepatic astrocytes [15]. Nevertheless, studies on the exploration of NREP in tumorigenesis is largely limited. In gastric cancer [10, 16], NREP promotes cell proliferation, angiogenesis, and EMT process. In human glioma cells, NREP inhibition decreased cell migration [17]. In lung cancer cell A549, NREP could promote cell cycle progression [18]. These findings indicate that NREP may play an important role in tumor cell proliferation and migration. However, its role in BC is still unknown.

Tumor cells mainly depend on glycolysis to produce energy [19–21]. Therefore, a targeted regulation of glycolysis may provide a therapeutic direction for BC treatment. Reported studies displayed that NREP could activate TGF-β1 [22], which promotes the malignant progression of tumor cells by inducing glycolysis through metabolic reprogramming [23, 24]. Also, NREP activates Rho GTPases family proteins like RhoA, Rac1, and RalA [25–27]. It has been proved that inhibition of Rac1 inhibits glycolysis and proliferation of BC cells [28]. Interestingly, BC is proved to have a considerably lower oxygen partial pressure than that in normal breast tissue [29], implying a hypoxic microenvironment of BC. Under hypoxia conditions, hypoxia-inducible factor HIF-1α promotes glycolysis of tumor cells, including BC. In addition, HIFs could activate the transcription of downstream factors, thereby participating in tumor progression [30]. Importantly, bioinformatics analysis showed that NREP is upregulated in hypoxia BC cells than in normoxia. Therefore, we speculated that NREP may be upregulated by HIF-1α, and involved in cellular glycolysis progression of BC.

In this study, we aimed to investigate the biological function role of NREP in proliferation and metastasis of BC, and the potential up-/down-stream regulatory mechanisms involved.

## Results

### NREP is upregulated and predicts poor prognosis value in BC clinical samples

**Fig. 1:**
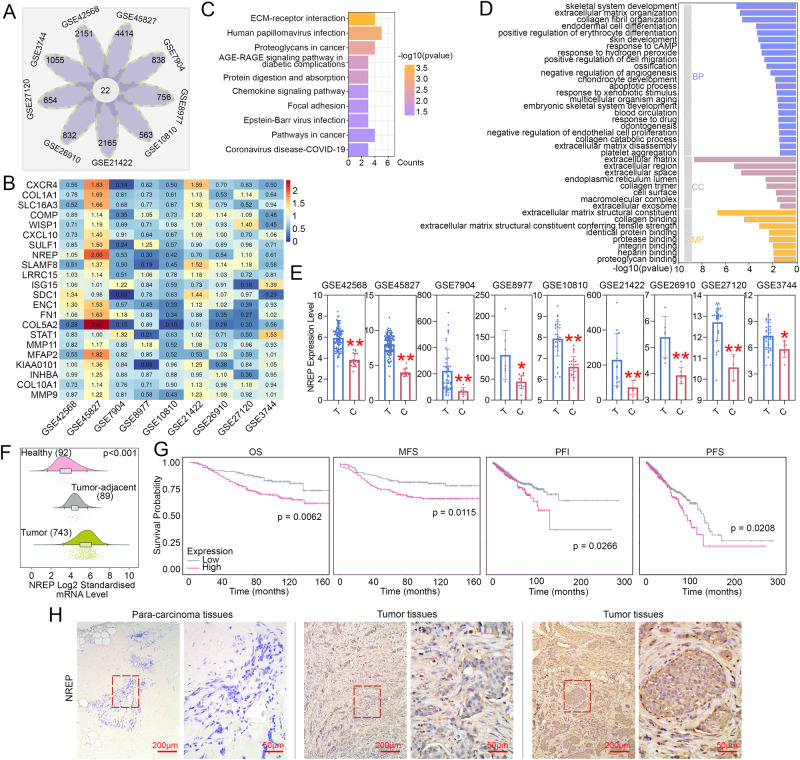
NREP is upregulated and predicts poor prognosis value in BC patients. **A** Venn diagram of 22 upregulated differentially expressed genes (DEGs) in nine BC databases from GEO. **B** Heat map of 22 DEGs in nine BC databases. **C** Kyoto Encyclopedia of Genes and Genomes (KEGG) enrichment analysis of 22 DEGs was analyzed by DAVIVD. **D** Gene ontology (GO) enrichment analysis of 22 DEGs was analyzed by DAVIVD. BP biological process, CC cellular component, MF molecular function. **E** The mRNA expression level of NREP in nine BC databases. T: tumor tissues; C: normal tissues. **F** The expression of NREP in the tumor, para-carcinoma, and healthy tissues of BC patients (analyzed by Breast Cancer Gene-Expression Miner v4.9). **G** Survival curves of BC patients with high or low NREP level (analyzed by Biomedical Informatics Institute). Overall survival (OS), metastasis-free survival (MFS), progression-free interval (PFI), and progression-free survival (PFS). **H** IHC staining of BC clinical samples with low or high NREP expression. Data were expressed as mean ± SD. *P < 0.05, **P < 0.01.

To explore the genes that may play an important role in promoting BC progression, we used 9 public available BC databases from GEO to screen the differentially expressed genes (DEGs). Venn diagram showed that there were 22 overlapping upregulated DEGs (Fig. 1A). The specific expression level of these 22 genes in each database was displayed as the heat map (Fig. 1B). Then, we used functional enrichment analyses to explore the function of these genes. For KEGG (Fig. 1C), these genes were related to the terms named “chemokine signaling pathway”, and “pathways in cancer”; and for GO (Fig. 1D), these genes were related to the terms named “positive regulation of cell migration”, “apoptotic process”, and “identical protein binding”. Among these genes, we noticed NREP, which exhibited a relatively higher expression level in all of these 9 databases. Compared with the normal tissues, tumor tissues exhibited a significantly higher expression level of NREP in these 9 databases (Fig. 1E). Besides that, NREP was also upregulated in BC tumor tissues as compared with the para-carcinoma and healthy tissues (Fig. 1F, analyzed by Breast Cancer Gene-Expression Miner v4.9). Survival curves showed that high-level NREP in BC patients displayed poor survival probability in overall survival (OS), metastasis-free survival (MFS), progression-free interval (PFI), and progression-free survival (PFS) (*p* < 0.05, Fig. 1G). Besides that, in BC patients, NREP exhibited higher expression in the tumor tissues when compared to the para-carcinoma tissues (Fig. 1H). Therefore, we suspected that the enhanced level of NREP may take a great part in BC tumor development.

### NREP is transcriptionally upregulated by HIF-1α in BC cells

**Fig. 2:**
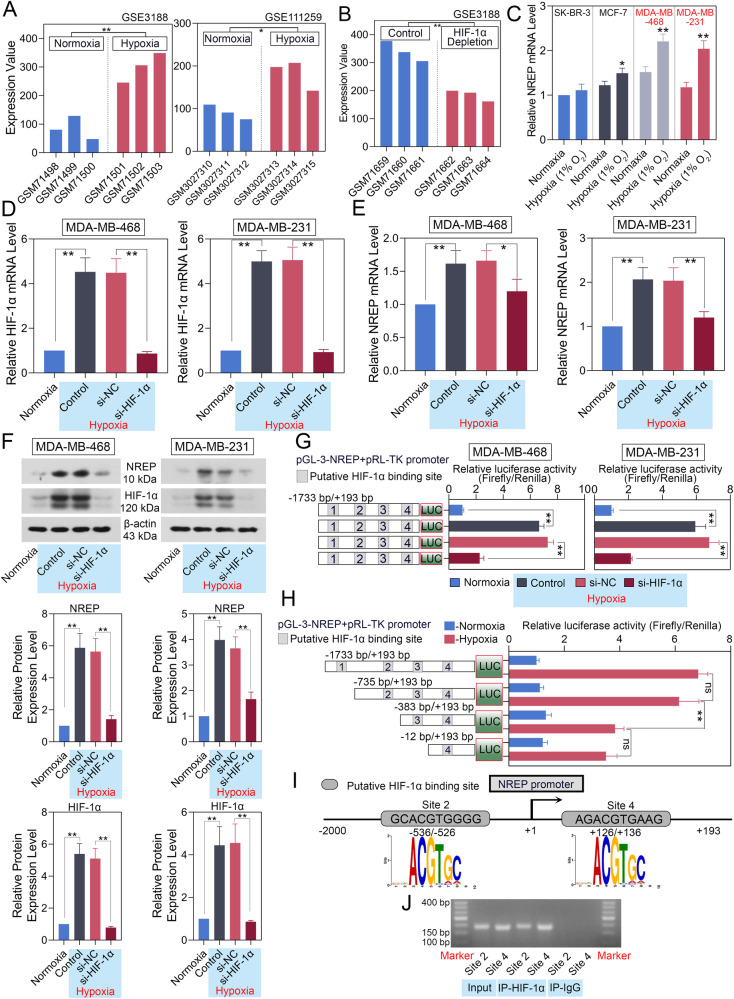
NREP is transcriptional activated by HIF-1α. **A** The expression of NREP in hypoxia and normoxia BC cells (data from GSE3188 and GSE111259). **B** The expression of NREP in HIF-1α depleted BC cells (data from GSE3188). **C** The mRNA expression of NREP in different BC cell lines (SK-BR-3, MCF-7, MDA-MB-468, MDA-MB-231) at normoxia or hypoxia was measured by qPCR. The mRNA expression of HIF-1α (**D**) and NREP (**E**) in HIF-1α inhibited MDA-MB-468 or MDA-MB-231 cells were measured by qPCR. **F** The protein expression and quantification data of NREP and HIF-1α in MDA-MB-468 or MDA-MB-231 cells were measured by western blot. **G**, **H** Luciferase reporter assay was used to confirm the binding sites of HIF-1α on NREP promoter. **I**, **J** ChIP-PCR assay was used to confirm that HIF-1α could directly bind with the promoter of NREP. Data were expressed as mean ± SD. *P < 0.05, **P < 0.01.

From GEO databases (GSE3188 and GSE111259), we found that NREP is upregulated in hypoxia BC tumor cells when compared to normoxia (Fig. 2A). Depletion of HIF-1α in BC cells exhibited an inhibition level of NREP (data from GSE3188, Fig. 2B). Then, we tested the expression of NREP in BC cell lines SK-BR-3, MCF-7, MDA-MB-468, and MDA-MB-231 (Fig. 2C). MDA-MB-468 and MDA-MB-231 showed a more obvious change between normoxia and hypoxia conditions. Thus, these two cell lines were chosen to achieve NREP up- or downregulation. Consist with the results from GEO databases, HIF-1α and NREP showed an increased mRNA (Fig. 2D, E) and protein (Fig. 2F) expression in hypoxia than normoxia, while downregulation of HIF-1α reversed this enhancement in BC cell lines. These results confirmed that hypoxia affects the expression of NREP in BC cells.

To verify if NREP could be directly regulated by HIF-1α, we used a dual-luciferase reporter system and ChIP-PCR assay for confirmation. In Fig. 2G, hypoxia caused a significantly increased transcriptional activity of the NREP promoter, while the downregulation of HIF-1α decreased this enhancement. In Fig. 2H, serial deletion results revealed the region between −735 to −383 bp (site 2) was essential to NREP-induced expression of luciferase reporter (significantly decreased luciferase activity). However, the regions between −1733 to −735 bp (site 1) and −383 to −12 bp (site 3) exhibited almost the same relative luciferase activity, implying that the putative HIF-1α binding sites in these two regions were unimportant. Besides that, the region between −12 to +193 bp (site 4) still has a high relative luciferase activity, suggesting that this site may also be important. Therefore, we designed primers for sites 2 and 4 (Fig. 2I) and performed a ChIP-PCR experiment. In Fig. 2J, protein bands appeared at sites 2 and 4 after immunoprecipitation. IgG used as a negative control exhibited no bands. This result confirmed that HIF-1α could directly bind to the promoter of NREP, thus upregulating its expression.

### NREP promotes BC cell growth and angiogenesis

**Fig. 3:**
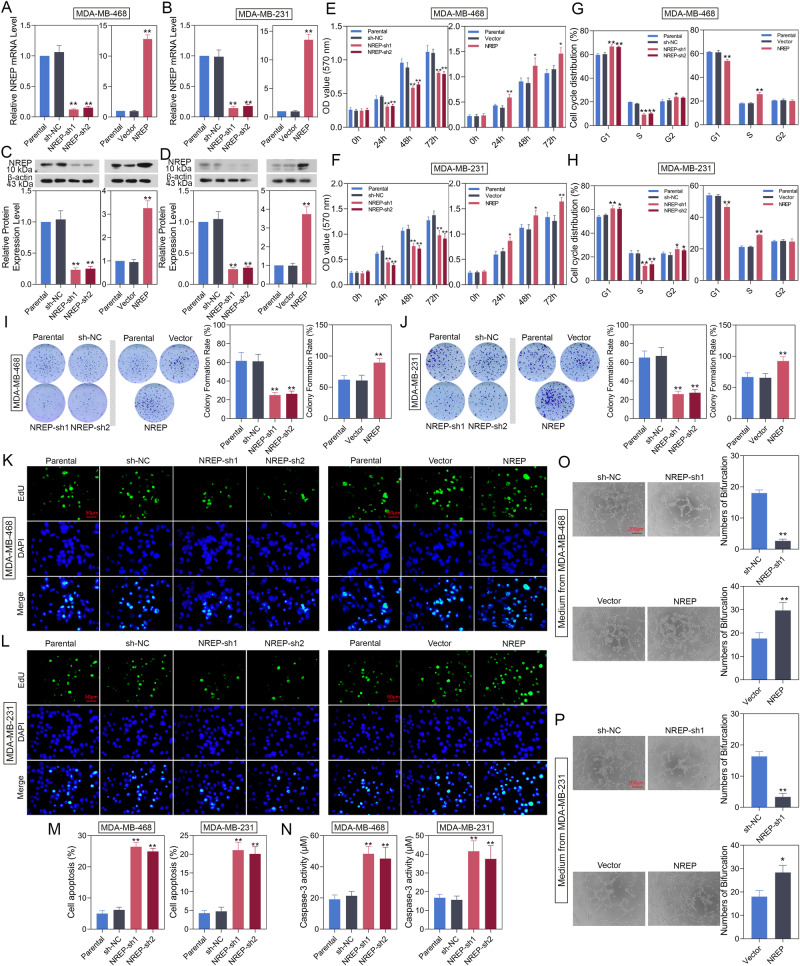
NREP promotes BC cell proliferation and angiogenesis in vitro. NREP was down-/up-regulated in MDA-MB-468 and MDA-MB-231 cells. **A**, **B** The mRNA expression of NREP in MDA-MB-468 or MDA-MB-231 cells was measured by qPCR. **C**, **D** The protein expression of NREP in MDA-MB-468 or MDA-MB-231 cells was measured by western blot. **E**, **F** Cell proliferation was analyzed by MTT assay in MDA-MB-468 or MDA-MB-231 cells. **G**, **H** The cell cycle was analyzed by flow cytometry in MDA-MB-468 or MDA-MB-231 cells. **I**, **J** Colony formation images and quantification data in MDA-MB-468 or MDA-MB-231 cells. **K**, **L** EdU staining images (green light) of MDA-MB-468 cells or MDA-MB-231 cells. DAPI: blue. **M** Cell apoptosis was analyzed by flow cytometry in MDA-MB-468 or MDA-MB-231 cells. **N** Caspase-3 activity in MDA-MB-468 or MDA-MB-231 cells. **O**, **P** Tube formation images and quantification data of HUVECs in tumor-conditioned medium. Data were expressed as mean ± SD. *P < 0.05, **P < 0.01.

NREP was down- or up-regulated in MDA-MB-468 and MDA-MB-231 cells, and mRNA (Fig. 3A, B) and protein expression (Fig. 3C, D) were verified. Then, we explored the effect of NREP on BC cell proliferation and apoptosis. Results showed that both in MDA-MB-468 and MDA-MB-231 cells, NREP knockdown suppressed cell viability (Fig. 3E, F), blocked the G1 to S phase transition in the cell cycle (Fig. 3G, H), decreased colony formation rate (Fig. 3I, J) and EDU positive cells (Fig. 3K, L), while all these changes were reversed in NREP overexpressed cells. In addition, NREP silencing increased the cell apoptosis rate (Fig. 3M) and caspase-3 activity (Fig. 3N) in MDA-MB-468 or MDA-MB-231 cells. Moreover, we investigated the effect of NREP on angiogenesis. HUVECs were cultured in tumor-conditioned medium (TCM) from NREP up- or down-regulated MDA-MB-468 and MDA-MB-231 cells. NREP silencing inhibited tube-like structure formation in HUVECs, while NREP overexpression induced it (Fig. 3O, P).

### NREP promotes BC cell migration, invasion and metastasis

**Fig. 4:**
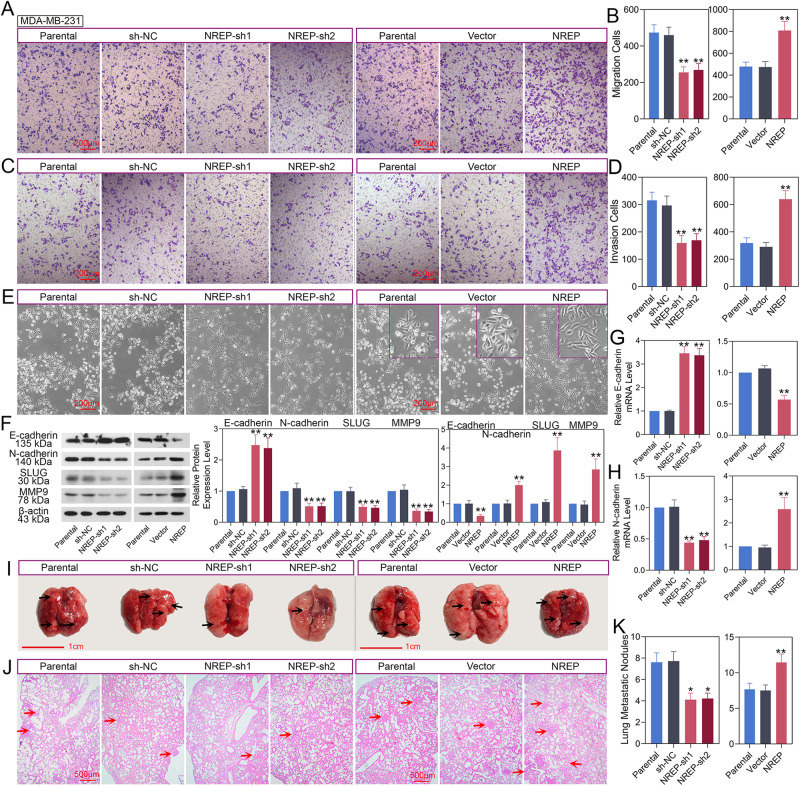
NREP promotes BC cell migration, invasion, and metastasis. **A**, **B** Cell migration was measured by transwell assay in NREP down-/up-regulated MDA-MB-231 cells. **C**, **D** Cell invasion was measured by transwell assay in NREP down-/up-regulated MDA-MB-231 cells. **E** The morphological changes of MDA-MB-231 cells were observed under a light microscope. **F** The protein expression and quantification data of E-cadherin, N-cadherin, SLUG, and MMP9 in NREP down-/up-regulated MDA-MB-231 cells were analyzed by western blot. **G**, **H** The mRNA expression of E-cadherin and N-cadherin in NREP down-/up-regulated MDA-MB-231 cells was analyzed by qPCR. **I** The images of metastasis lesions in mouse lung tissues. **J**, **K** H&E staining images and quantification data of metastasis lesions in mouse lung tissues. Data were expressed as mean ± SD. *P < 0.05, **P < 0.01.

After that, we explored the effect of NREP on BC metastasis in MDA-MB-231 cells. Images and quantification data from the Transwell assay demonstrated that knockdown of NREP inhibited the numbers of migrated (Fig. 4A, B) and invaded (Fig. 4C, D) cells while overexpressing NREP promoted it. In addition, MDA-MB-231 cells exhibited striking morphological modifications. Upregulation of NREP triggered the transition from a cobblestone-like morphology to an elongated shape associated with increased cell scattering (Fig. 4E). Also, the E-cadherin expression was upregulated, and N-cadherin, SLUG, and MMP9 protein expression were decreased in response to NREP inhibition (Fig. 4F). Meanwhile, the mRNA expression of E-cadherin was upregulated (Fig. 4G), and N-cadherin was downregulated (Fig. 4H) with NREP knockdown. In NREP upregulated MDA-MB-231 cells, all these trends were reversed.

Then, we analyzed the effect of NREP on BC metastasis in vivo. In Fig. 4I, the metastatic nodules in the lungs of mice injected with NREP-knockdown cells were less than that of mice injected with control cells, while more metastatic nodules were apparent in mice injected with NREP-overexpression cells than of vector infected cells. Similarly, the H&E staining images of lung tissues (Fig. 4J) and quantification data of metastatic nodules (Fig. 4K) also showed the same result.

### NREP promotes BC tumor growth in vivo

**Fig. 5:**
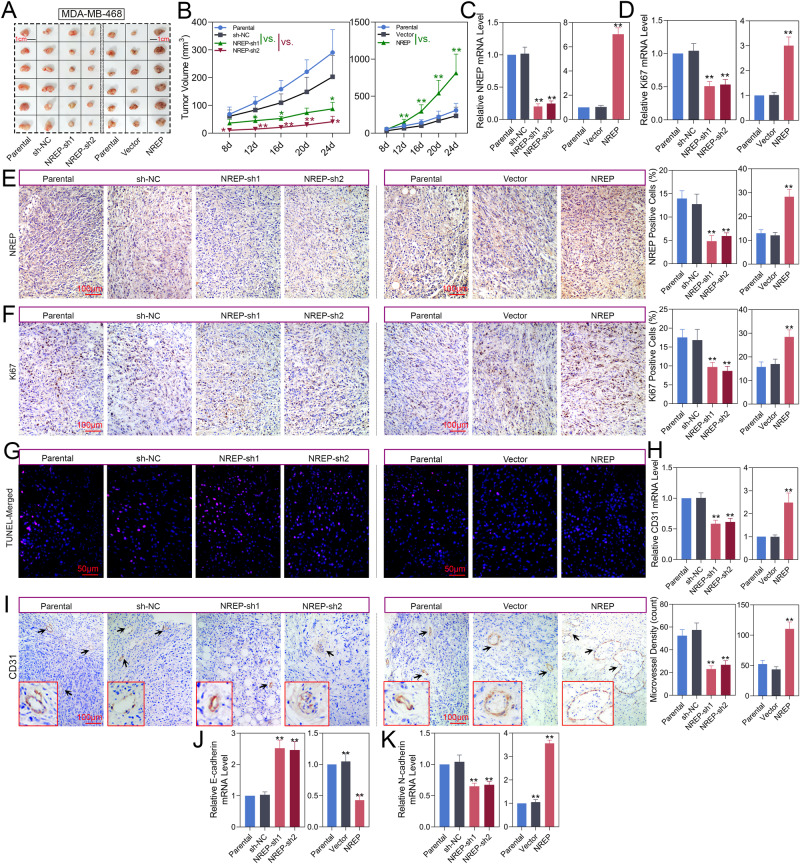
NREP promotes BC tumor growth in vivo. **A** Tumor image of xenograft mice injected with NREP-shRNA or -OE infected MDA-MB-468 cells. **B** Tumor volume changes in the nude mice that were injected with NREP-shRNA or -OE infected MDA-MB-468 cells were analyzed over time. **C**, **D** The mRNA expression of NREP and Ki67 in mice formed tumor tissues was analyzed by qPCR. **E**, **F** The IHC images and positive cells of NREP and Ki67 in mice formed tumor tissues. **G**, **H** TUNEL staining images of mice formed tumor tissues. **I** IHC images of CD31 and microvessel density of mice formed tumor tissues. **J**, **K** The mRNA expression of E-cadherin and N-cadherin in mice formed tumor tissues was analyzed by qPCR. Data were expressed as mean ± SD. *P < 0.05, **P < 0.01.

Based on these results, we confirmed the promotion effect of NREP on BC progression in vivo. Mice were injected with NREP-sh or -ove infected MDA-MB-468 cells. The images and volume changes of tumors were shown in Fig. 5A, B. Similar to the in vitro result, the knockdown of NREP decreased tumor growth, while overexpression of NREP showed an opposite result that promotes tumor growth. The decrease of NREP (Fig. 5C) and Ki67 (Fig. 5D) mRNA expression, fewer NREP-positive cells (Fig. 5E) and Ki67-positive cells (Fig. 5F), and an increase of red fluorescence of TUNEL images (Fig. 5G) were observed in NREP silenced cells formed tumor tissues. Besides that, suppressed CD31 mRNA level (Fig. 5H), decreased microvessel density (Fig. 5I), increased E-cadherin mRNA level (Fig. 5J), and decreased N-cadherin mRNA level (Fig. 5K) were also observed in NREP silenced cell formed tumor tissues. In NREP upregulated cells formed tumor tissues, and all these trends were reversed.

### NREP accelerates cellular glycolysis in BC cells

**Fig. 6:**
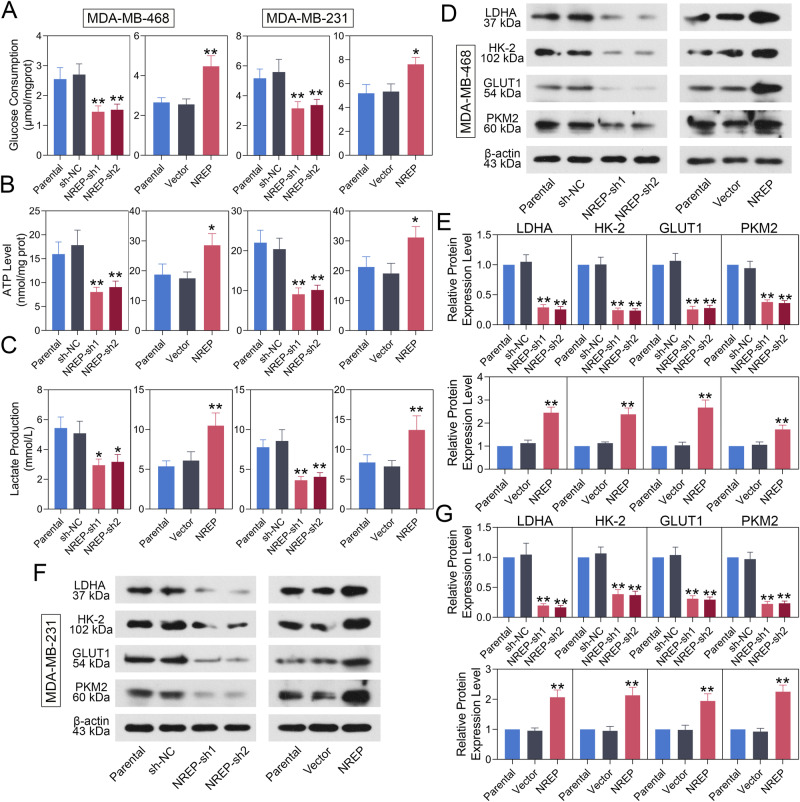
NREP promotes glycolysis in BC cells. Glucose consumption (**A**), ATP level (**B**), and lactate production (**C**) in NREP down-/up-regulated MDA-MB-468 or MDA-MB-231 cells. **D**, **E** The protein expression and quantification data of LDHA, HK2, GLUT1, and PKM2 in NREP down-/up-regulated MDA-MB-468 cells. **F**, **G** The protein expression and quantification data of LDHA, HK2, GLUT1, and PKM2 in NREP down-/up-regulated MDA-MB-231 cells. Data were expressed as mean ± SD. *P < 0.05, **P < 0.01.

After that, we sought to determine the potential role of NREP in the glucose metabolism of BC. As shown in Fig. 6A–C, either in MDA-MB-468 or MDA-MB-231, NREP regulation displayed the same trend. NREP knockdown decreased cellular glucose consumption, ATP level, and lactate production, while upregulation of NREP led to increased levels in BC cells. Then, we detected the protein expression of glucose transporters in BC cells. As exhibited, NREP inhibition had a lower protein level of GLUT1, HK2, PKM2, and LDHA in MDA-MB-468 (Fig. 6D, E) or MDA-MB-231 (Fig. 6F, G) cells. Conversely, in NREP upregulated cells, all of these genes were upregulated. Therefore, these results indicated that NREP may promote the growth and metastasis of BC by regulating cellular glycolysis.

### RNA-seq and functional enrichment analyses of NREP silenced BC cells

**Fig. 7:**
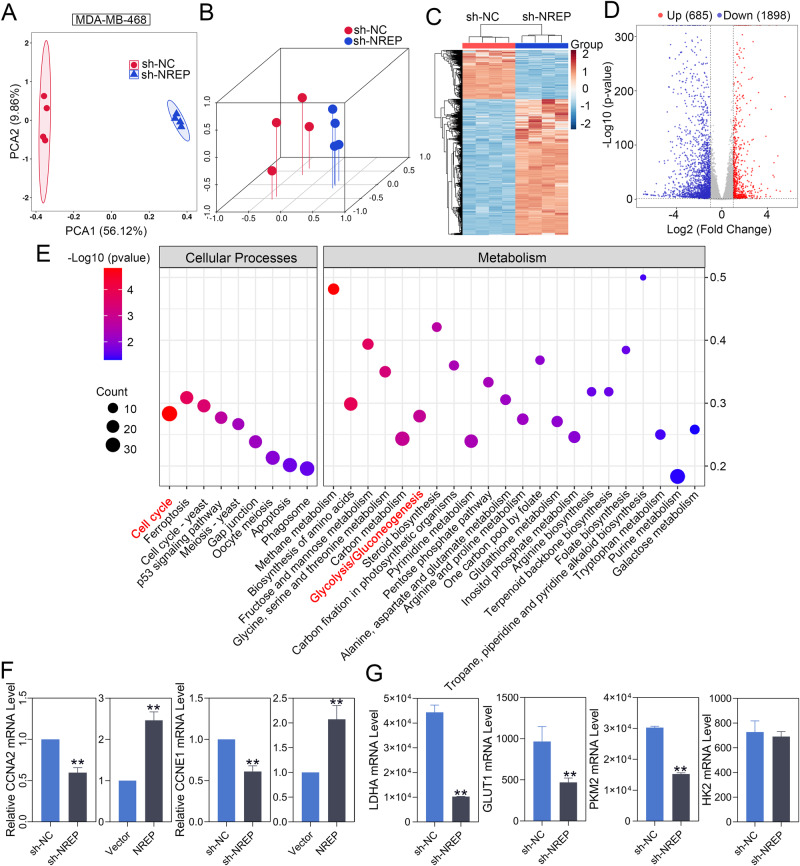
RNA-seq and functional enrichment analyses in NREP silenced BC cells. **A**, **B** Principal component analysis (PCA) plot of sh-NC and sh-NREP infected MDA-MB-468 cells. **C** Hierarchical clustering analysis was performed based on all DEGs. **D** Volcano map of all DEGs. The red dot stands for upregulated DEGs, blue dot stands for downregulated DEGs. **E** Selected KEGG enrichment pathway of all DEGs. **F** The mRNA expression of CCNA2 and CCNE1 in NREP knockdown or overexpressed MDA-MB-468 cells. **G** The mRNA expression of LDHA, GLUT1, PKM2, and HK2 in NREP suppressed MDA-MB-468 cells (Data from RNA-seq). Data were expressed as mean ± SD. *P < 0.05, **P < 0.01.

After that, RNA-seq was used in NREP-inhibited MDA-MB-468 cells to explore the downstream factors and pathways of NREP. PCA plot (Fig. 7A, B) and hierarchical clustering plot (Fig. 7C) were performed to confirm the consistency and variance of samples. Volcano map exhibited all DEGs, including 685 upregulated DEGs and 1898 downregulated DEGs (Fig. 7D). Then, we performed the GO and KEGG functional enrichment analyses in all DEGs. The top 20 terms of GO were mainly enriched in “protein binding”, “cytosol” and “cytoplasm” (Fig. S1A). As for KEGG, a total of 51 pathways were enriched (*p* < 0.05). The pathways that related to “Cellular Processes” and “Metabolism” were exhibited in Fig. 7E, and the others were shown in Fig. S1B. Then, the DEGs that enriched in “cell cycle” and “glycolysis/gluconeogenesis” pathways were focused. We confirmed the effect of NREP regulation on CCNA2 and CCNE1 expression, both of them played an important role in the cell cycle G1/S transition. Downregulation of NREP suppressed the mRNA expression of CCNA2 and CCNE1 in MDA-MB-468 cells, while overexpressing of NREP promoted their expression (Fig. 7F). Besides that, based on RNA-seq results, we showed the mRNA expression of glycolysis-related factors. Downregulation of NREP suppressed the mRNA expression of LDHA, GLUT1, and PKM2 in MDA-MB-468 cells (Fig. 7G).

## Discussion

As a malignant disease threatening women’s health, BC has always been the focus of the majority of studies in cancer. In this work, we found that NREP is highly expressed in BC tumor tissues and cell lines. HIF-1α as an upstream factor transcriptionally activated NREP and upregulated its expression. Highly expressed NREP promoted cell proliferation, angiogenesis, migration, invasion, and EMT process of BC cells in vitro and in vivo. Moreover, NREP promoted glycolysis of BC cells. Accordingly, we demonstrated that aberrantly expressed NREP may play a promoting role in tumor growth and metastasis in BC by promoting cell proliferation and glycolysis.

Hypoxia is an essential factor in cancer development [31–34], and this effect was related to the activation of downstream genes that are subject to HIF-1α [35–37]. In BC cells, HIF-1α plays a critical role in stimulating the metastasis [37] of primary tumor to distant organs, which is closely related to patient mortality. Knockdown of HIF-1α abrogates this effect of hypoxia and significantly impairs the tumor-initiating ability of BC cells [31]. These findings indicated that hypoxia is essential for BC tumor development. Therefore, after confirming that NREP is highly expressed in different BC databases and that its expression may be induced by hypoxia, we first verified whether NREP functions as downstream of HIF-1α. Consistent with online analysis, our results showed that knockdown of HIF-1α decreased the level of NREP in BC cells. Dual luciferase reporter and ChIP-PCR assay confirmed that HIF-1α could bind to the promoter of NREP, thus regulating its expression. Therefore, we referred that the enhanced level of NREP may take a great part in BC tumor development, and this process may be related to HIF-1α.

After that, we explore the effect of NREP on the malignant phenotype of BC cells in vitro and in vivo. So far, the function of NREP in gastric cancer has been explored, and they found that NREP promotes the growth and metastasis of gastric cancer [10, 16]. Similar to the reported study [10, 16], upregulation of NREP promotes cell proliferation, angiogenesis, migration, invasion, and EMT process in vitro and in vivo. These findings indicated that NREP played an oncogene role in BC tumor growth and metastasis. In lung cancer cell A549, NREP could promote the G1/S phase transition of the cell cycle [18]. Therefore, we examined the effect of NREP on the BC cell cycle. Results showed that NREP downregulation blocked the G1 to S phase transition in the cell cycle, while overexpressed NREP increased the S phase, and decreased the G1 phase. Besides that, NREP restrains cell apoptosis in BC cells, which is similar to its role in gastric cancer cell apoptosis [10]. These findings help to explain the promotion effect of NREP in BC cell proliferation.

After that, we explored the potential mechanism of NREP in BC. In this work, we confirmed that the expression of NREP could be upregulated by HIF-1α. As we know, HIF-1α is the master regulator of glucose metabolism [38]. Additionally, NREP could activate TGF-β1 [22] and Rac1 [25–27]. TGF-β1 could promote malignant progression of tumor cells by inducing glycolysis [23, 24], and inhibition of Rac1 inhibits glycolysis and proliferation of BC cells [28]. Therefore, we suspected that NREP, which is upregulated by HIF-1α, may affect glycolysis of BC cells. As we mentioned before, glycolysis is a distinctive cellular metabolic manner in cancer cells exhibiting an increasing rate of glucose uptake and lactic acid fermentation. Therefore, we detected the effect of NREP on glycolysis-related factors. NREP upregulation increased cellular glucose consumption, ATP level, and lactate production. Besides that, NREP promoted the expression of glucose transporters GLUT1, HK2, PKM2, and LDHA in BC cells. Therefore, these results indicated that NREP may promote the growth and metastasis of BC by modulating glycolysis. However, due to the unavailability of the instrument, extracellular acidification rate (ECAR) detection was missed, which is considered a limitation of our current research.

Moreover, considering that the function of NREP in tumors is less explored, for further exploration, we used RNA-seq to explore the genes that may be regulated by NREP. In KEGG functional analysis, the DEGs that enriched in “glycolysis/gluconeogenesis” and “cell cycle” pathways were focused. With NREP knockdown, 19 DEGs were enriched in the “glycolysis/gluconeogenesis” term. Through the published studies, we confirmed the promotion (LDHA [39], PGAM4 [40], ACSS2 [41], PGAM1 [42, 43], ENO2 [44], ALDOA [45], BPGM [46], PFKL [47], HK1 [48], PGM2 [49], ALDOC [50], PGK1 [51], PFKP [52], TPI1 [53, 54], and ALDH3B1 [55]) or inhibition (ALDH2 [56], PCK2 [57], and FBP2 [58, 59]) functions of these genes on glycolysis, and the result confirmed that NREP affects glycolysis of BC cells. Besides that, LDHA, GLUT1, and PKM2 were significantly downregulated in NREP-silenced BC cells. Thus, it is reasonable to believe that NREP is at least partially involved in HIF-1a induced expression of glycolysis-related genes. Besides that, overexpressing of NREP promoted the expression of CCNA2 and CCNE1, which is consistent with its function on the cell cycle.

Apart from cell cycle and glycolysis, “DNA replication” pathway was also significantly enriched in KEGG analysis. This result suggested that NREP may also play a carcinogenic role in BC by affecting the factors that related to DNA replication. In addition, based on RNA-seq results, the mRNA expression of the top 10 down-regulated DEGs (PNMT, SMIM10L2B, SNCB, SLC30A3, PLCB2, PTPRN, ADGRE1, GABRD, NTNG2, and BATF3) was validated in NREP up-/down-regulated MDA-MB-468 cells, and the results confirmed that NREP regulates the expression of these genes (Figure S2). Among these genes, SLC30A3 [60], PLCB2 [61], PTPRN [62, 63], GABRD [64, 65], and BATF3 [66] have been reported were involved in cell proliferation, migration, or metastasis in tumors. PLCB2 is highly expressed in BC and is associated with a poor outcome [67]. Thus, these genes may also be involved in NREP mediated malignant phenotypes of BC progression. Of course, more experiments were needed for verification, and these factors and pathways will also be a key direction for our subsequent exploration of NREP.

Taken together, our study showed evidence that NREP could be transcriptionally activated by HIF-1α. In addition, NREP may promote BC tumor growth and metastasis by modulating glycolysis. In summary, the above findings illustrate the possibility that NREP takes great part in BC progression.

## Materials and methods

### Data collection and analyses

Expression data of BC datasets GSE42568 (17 normal, 104 tumors), GSE45827 (11 normal, 130 tumors), GSE7904 (7 normal, 43 tumors), GSE8977 (15 normal, 7 tumors), GSE10810 (27 normal, 31 tumors), GSE21422 (5 normal, 14 tumors), GSE26910 (6 normal, 6 tumors), GSE27120 (3 normal, 28 tumors), GSE3744 (7 normal, 40 tumors), GSE3188 (BC cells in normoxia or hypoxia condition), and GSE111259 (BC cells in normoxia or hypoxia condition) were downloaded from Gene Expression Omnibus (GEO) and analyzed by GEO2R (https://www.ncbi.nlm.nih.gov/geo/geo2r/). Upregulated differentially expressed genes (DEGs) were selected with Log_2_Foldchange > 1 and p < 0.05. Gene ontology (GO) and the Kyoto Encyclopedia of Genes and Genomes (KEGG) were analyzed by DAVID (https://david.ncifcrf.gov/). NREP expression in BC tumor tissues, para-tumor, and healthy tissues was analyzed by Breast Cancer Gene-Expression Miner v4.9 (http://bcgenex.ico.unicancer.fr/BC-GEM/GEM-Accueil.php?js=1). The survival curve was analyzed by the Biomedical Informatics Institute (https://bioinfo.henu.edu.cn/Index.html).

### Clinical sample collection

BC clinical patients were collected for IHC staining. All specimens were collected according to the provisions of the Declaration of Helsinki, and approved by the Ethics Committee of Zhengzhou University.

### Cell culture and treatment

All BC cell lines were obtained from iCell (China). SK-BR-3 cells were cultured in McCoy’s 5 A medium (PM150710, Procell, China), MCF-7 cells were cultured in MEM (41500, Solarbio, China), MDA-MB-468 and MDA-MB-231 cells were cultured in L15 (PM151011, Procell, China). All mediums were added with 10% fetal bovine serum (FBS, 11011-8611, Tianhang Biotech, China), and cells were incubated with 5% CO_2_ at 37 °C. For hypoxia, cells were placed in a hypoxia chamber maintained at 1% oxygen for 24 h. All cell lines have passed short tandem repeat (STR) profiling and cultured free from mycoplasma contamination.

Lentiviral infection was carried out for NREP regulation. For the knockdown of NREP, vectors of two short hairpin RNAs (shRNA) targeting NREP mRNA and control vector (pLVX-shRNA1, VT1456, Youbio Biotech, China) were used. Sequences specific targeting the NREP: shNREP-1 (5ʹ-AGAAGAACGATGAGACAAACG-3ʹ) and shNREP-2 (5ʹ-CAAAGGAAGTGAACCGCAAGA-3ʹ). For overexpression of NREP, the pLVX-IRES-puro vector (Youbio Biotech, China) was used. Stably infected clones were obtained through puromycin screening. For cell transfection, Lip3000 (L3000015, Invitrogen, USA) was used.

### Real-time quantitative PCR (qPCR)

Total RNA was extracted from cultured cells by TRIpure (RP1001, BioTeke, China). Concentration was determined by an ultraviolet spectrophotometer (NANO 2000, Thermo, USA). The cDNA was obtained by RNase inhibitor (RP5602, BioTeke, China) and BeyoRT II M-MLV reverse transcriptase (D7160L, Beyotime, China). The mRNA levels of indicated genes were quantified by qPCR using 2×Taq PCR MasterMix (PC1150, Solarbio, China) and SYBR Green (SY1020, Solarbio, China) in PCR instrument (Exicycler^TM96^, BIONEER, Daejeon, Korea). Expression levels were calculated using the 2^−ΔΔCt^ method and normalized to that of β-actin. Primer sequences used for qPCR are shown in Table S1.

### Western blotting

Cells were lysed by PMSF added lysate (ST506 and P0013, Beyotime, China). Protein concentration was measured by a BCA detection kit (P0011, Beyotime, China). Then, the protein was subjected to SDS-polyacrylamide gel electrophoresis (P0015, Beyotime, China) and transferred to polyvinylidene fluoride (PVDF) membranes (IPVH00010, Millipore, USA). After blocked with 5% nonfat milk (YiLi, China) for 1 h at room temperature, the membrane was incubated with primary antibody overnight at 4 °C, followed by horseradish peroxidase (HRP)-conjugated goat anti-rabbit (A0208, Beyotime, China) or anti-mouse (A0216, Beyotime, China) antibody at 1:5000 dilution for 45 min. The blots were incubated in ECL substrate (P0018, Beyotime, China), and images were analyzed using the Gel-Pro-Analyzer software.

Antibody information: NREP (1:500, PA5-68426, Thermofisher, USA), HIF-1α (1:500, AF1009, Affinity, China), E-cadherin (1:1000, A20798, Abclonal, China), N-cadherin (1:500, A19083, Abclonal, China), SLUG (1:1000, A1057, Abclonal, China), MMP9 (1:500, A0289, Abclonal, China), PKM2 (1:500, A13905, Abclonal, China), GLUT1 (1:1000, AF5462, Affinity, China), HK-2 (1:500, A20829, Abclonal, China), and LDHA (1:500, A1146, Abclonal, China).

### Luciferase reporter assay

Dual luciferase reporter gene assay kit (KGAF040, KeyGEN BioTech, China) according to the manufacturer’s instruction. The NREP promoter region was inserted into the pGL3-Basic vector (BR014, Fenghui Bio, China) and co-transfected with pRL-TK, siNC, or si-HIF-1α into BC cells. HIF-1α siRNA was synthesized by General Biosystems (China). HIF-1α siRNA: 5ʹ-CAGAAAUGGCCUUGUGAAATT-3ʹ, 5ʹ-UUUCACAAGGCCAUUUCUGTT-3ʹ. After 24 h, BC cells were cultured in normoxia (N) or hypoxia (H) for another 24 h. After transfection, cells were treated with 250 μL lysis buffer. Then, 100 μL firefly luciferase detection reagent and 20 μL sample were added to each well, and the mixture was gently blown before reading. After that, 100 μL renilla luciferase detection reagent was added to each well. Luciferase activity was measured with a multifunctional microplate reader (M200Pro, TECAN, Switzerland). The relative luciferase activity was calculated by firefly/renilla.

### Chromatin immunoprecipitation (ChIP)

The ChIP detection kit (WLA106a, Wanlei Bio, China) was used according to the manufacturer’s instructions. Cells were treated with 1% formaldehyde to crosslink chromatin associated proteins to DNA. Cell lysates were subjected to ultrasound for 12 sets of 10 s (interval: 30 s) to obtain DNA fragments. Cell lysates were incubated with anti-HIF-1α (20960-1-AP, Proteintech, China), anti-IgG (negative control), and anti-RNA polymerase II (positive control). All the above chromatin supernatants were incubated with 60 μL magnetic protein A/G beads at 4 °C for 1–2 h with rotation. After that, the protein-DNA complexes were reversed and purified by a DNA Gel Extraction Kit (Wanlei Bio, China). DNA products were verified by PCR. Primer for site 2 was forward 5ʹ-TGACTTAGCCCACAGACACT-3ʹ and reverse 5ʹ-AAAGCAGAAGCCATGAATAT-3ʹ, and for site 4 was forward 5ʹ-AACTATCCACCATCTTG-3ʹ and reverse 5ʹ-ACACCACCTACCACTG-3ʹ.

### MTT assay

MTT detection kit (C0009, Beyotime, China) was used in this section. MTT solution (10 μL) was added to each well and the cells were incubated for 4 h. Formazan (100 μL) was added and continued to incubate until dissolved. The OD value at 570 nm was measured by a microplate reader (800TS, BIOTEK, USA).

### Cell cycle

Cell cycle distribution in BC cells was measured by a detection kit (KGA9101, KeyGEN BioTech, China). After centrifugation, cells were fixed in 70% ethanol overnight at 4 °C. Then, cells were washed and resuspended in 500 μL PI/RNase A working solution. Cells were incubated for 30 min in the dark, followed by flow cytometry detection (NovoCyte, Agilent, USA).

### Colony formation assay

Cells (400 per dish) were seeded and the colonies could be formed about 15 days later. Cells were fixed with 4% paraformaldehyde (C104188, Aladdin, China) for 25 min at room temperature. Then, Weigert-Giemsa solution (KGA227, KeyGen Biotech, China) was added and stained for 5 min. Colonies containing more than 50 cells were counted under a microscope. Colony formation rate = (number of colonies /number of inoculated cells) ×100%.

### EdU staining

EdU imaging detection kit (KGA331, KeyGen Biotech, China) was used in this section. Cells were treated with 10 μM EdU staining solution and incubated for 2 h at 37 °C. Then, cells were fixed with 4% paraformaldehyde for 15 min and 0.5% Triton X-100 for 20 min (0.1 mL) at room temperature. Finally, the click-it solution was added and incubated for 30 min in the dark. After stained with DAPI, images were taken under a fluorescence microscope (IX53, OLYMPUS, Japan).

### Tube formation assay in HUVECs

HUVEC cells were cultured in a dedicated medium (PriMed-iCell-002, iCell, China). Matrigel (30 µL; 356234, Corning, USA) was plated in 96-well plates for 2 h at 37 °C in the incubator. Then, HUVECs were seeded, and BC cell-derived conditioned medium was added into wells. Then, cells were cultured in an incubator with 5% CO_2_ at 37 °C. After 6 h, tubule formation was assessed using microscopy.

### Cell apoptosis

Apoptosis in BC cells was measured by Annexin V-FITC/PI double staining apoptosis detection kit (KGA1102, KeyGEN BioTech, China). After centrifugation, cells were resuspended in 500 μL binding buffer. Then, 5 μL AnnexinV-FITC and 5 μL PI were separately added and mixed. Cells were incubated for 15 min in the dark, followed by flow cytometry detection (NovoCyte, Agilent, USA).

### Animal experiments

Four weeks old female BALB/c mice (SPF, Cavens, China) were divided into the following groups: Parental, shNC, NREP-sh1, NREP-sh2, Parental, Vector, and NREP, based on the random number table approach. Six mice were included in each group to ensure the accuracy and completeness of the experimental results. The sample size was estimated by power analysis and our experience. No animals were excluded.

For each mouse, 5 × 10^6^ cells were subcutaneously injected into the groin. Tumor volumes were measured every 4 days after sizeable tumor formation. At the end of the experiment (24 days later), mice were sacrificed, and tumors were dissected and weighed. For tumor lung metastasis, the mouse was injected with 2.5 × 10^6^ cells via the tail vein. The mice were sacrificed after nine weeks, and the lungs were removed and collected for further analysis.

### Immunohistochemistry (IHC) assay

After deparaffinization and hydration, tissue sections (5 μm) were treated in antigen retrieval solution. Sections were then treated with 3% H_2_O_2_ (10011218, Sinopharm Chemical Reagent Co., Ltd., China) for 15 min to block endogenous peroxidase activity, and then incubated with 1% BSA (A602440-0050, Sangon Biotech, China) to block nonspecific antibody binding. Primary antibody was added to the sections at 4 °C overnight, followed by HRP-labeled goat anti-rabbit IgG (1:500, #31460, ThermoFisher, USA) at 37 °C for 1 h. Sections were then treated with DAB (DAB-1031, Maixin-Bio, China) and hematoxylin (H8070, Solarbio, China). Images were taken under a microscope (BX53, OLYMPUS, Japan). Antibody information: NREP (1:100, DF14359, Affinity, China), Ki67 (1:100, AF0198, Affinity, China), CD31 (1:100, A4900, Abclonal, China).

### TUNEL staining

Cell apoptosis in mice formed tumor tissues was measured by In Situ Cell Death Detection Kit (12156792910, Roche, Switzerland). Sections (5 μm) were added with 0.1% Triton X-100 (ST795, Beyotime, China) for 8 min at room temperature. Then, the TUNEL working solution was prepared and incubated for 1 h at 37 °C in the dark. DAPI (D106471-5mg, Aladdin, China) was used for counterstaining. Images were taken under a microscope (BX53, OLYMPUS, Japan).

### Transwell assay

Cell migration and invasion were measured by transwell assay. For cell migration, the transwell chamber (14341, LABSELECT, China) was placed into a 24-well plate, 800 µL of culture medium containing 10% FBS was added to the lower chamber, and 200 µL of cell suspension was added to the upper chamber, followed by 24 h incubation at 37 °C in the incubator with 5% CO_2_. For cell invasion, transwell chambers were placed in 24-well plates, coated with pre-diluted matrigel gel (356234, Corning, USA), and placed in an incubator at 37 °C for 2 h. After that, a transwell chamber containing matrigel gel was placed into a 24-well plate, 800 µL of culture medium containing 10% FBS was added to the lower chamber, and 200 µL of cell suspension was added to the upper chamber, followed by 48 h incubation. After washing, the chambers were fixed with 4% paraformaldehyde and stained with crystal violet staining solution (0528, Amresco, USA) for 5 min. The cells were counted under a microscope (IX53, OLYMPUS, Japan). Five random fields were selected from each sample, and the average was taken.

### H&E staining

Tissue sections (5 μm) were treated with hematoxylin (H8070, Solarbio, China) for 5 min, 1% hydrochloric acid alcohol for 3 s, running water for 20 min, and eosin staining solution (A600190, Sangon Biotech, China) for 3 min. After that, sections were successively immersed in different concentrations of ethanol (10009218, Sinopharm Chemical Reagent Co., Ltd., China) and xylene (1330-20-7, Aladdin, China). Images were taken under a microscope (BX53, OLYMPUS, Japan).

### Detection kits

Caspase 3 activity (C1116, Beyotime, China), glucose consumption (F006, Nanjing Jiancheng Bioengineering Institute, China), ATP level (S0026, Beyotime, China), and lactate production (A019, Nanjing Jiancheng Bioengineering Institute, China) were measured by different detection kits according to the manufactures’ instruction. Protein concentration was detected by BCA kit (P0011, Beyotime, China) or Bradford protein assay kit (P0006, Beyotime, China). Results were read by a microplate reader (ELX-800, BIOTEK, USA).

### RNA-sequencing (RNA-seq)

NREP downregulated MDA-MB-468 cells were cultured and collected for RNA-seq to analyze the downstream genes that may be regulated by NREP. RNA integrity was assessed using the Fragment Analyzer 5400 (Agilent Technologies, CA, USA). DEGs were selected with |Log_2_Foldchange | > 1 and *p* < 0.05.

### Statistical analysis

All evaluators were blinded to the assignments, and all data were used for analysis. Data were shown as mean ± standard deviation (S.D.). Statistical analysis was performed using GraphPad 8.0 statistical software. Normality and homogeneity of variance were tested at first. For comparison between the two groups, an unpaired t-test was employed according to the homoscedasticity of variances, and Mann Whitney test was applied to the non-normally distributed data. For comparisons among multiple groups, one-way ANOVA followed Tukey’s multiple comparisons test was applied to the normally distributed and homogeneous data, and Kruskal-Wallis’s test was applied to the non-normally distributed data. *P* values less than 0.05 were considered statistically significant.

### Supplementary information

Supplementary data-Revised

Supplementary material-Western Blot

## Data Availability

The authors will provide data included in the present research upon request.

## References

[1] Butera A, Cassandri M, Rugolo F, Agostini M, Melino G (2020). The ZNF750-RAC1 axis as potential prognostic factor for breast cancer. Cell Death Discov..

[2] Dewangan J, Srivastava S, Mishra S, Divakar A, Kumar S, Rath SK (2019). Salinomycin inhibits breast cancer progression via targeting HIF-1α/VEGF mediated tumor angiogenesis in vitro and in vivo. Biochem Pharmacol.

[3] Islam SS, Al-Tweigeri T, Al-Harbi L, Ujjahan S, Al-Mozaini M, Tulbah A (2024). Long noncoding RNA DLEU2 and ROR1 pathway induces epithelial-to-mesenchymal transition and cancer stem cells in breast cancer. Cell Death Discov..

[4] Dewangan J, Kaushik S, Rath SK, Balapure AK (2018). Centchroman regulates breast cancer angiogenesis via inhibition of HIF-1α/VEGFR2 signalling axis. Life Sci..

[5] Galluzzi L, Vitale I, Aaronson SA, Abrams JM, Adam D, Agostinis P (2018). Molecular mechanisms of cell death: recommendations of the Nomenclature Committee on Cell Death 2018. Cell Death Differ..

[6] Jiao X, Wang B, Yang L, Zhao Q, Zhang M, Liu X (2022). FMNL2 suppresses cell migration and invasion of breast cancer: a reduction of cytoplasmic p27 via RhoA/LIMK/Cofilin pathway. Cell Death Discov..

[7] Liu Z, Yang J, Chen Y, Chen C, Wang J, Lee YM (2022). P311 facilitates the angiogenesis and wound healing function of MSCs by increasing VEGF production. Front. Immunol..

[8] Stradiot L, Mannaerts I, van Grunsven LA (2018). P311, friend, or foe of tissue fibrosis?. Front. Pharmacol..

[9] Nunez S, Young C, Adebayo O, Muppuru KM, Badri KR (2019). P311, a novel intrinsically disordered protein, regulates adipocyte development. Biochem Biophys Res Commun..

[10] Liu YJ, Zeng SH, Hu YD, Zhang YH, Li JP (2021). Overexpression of NREP promotes migration and invasion in gastric cancer through facilitating epithelial-mesenchymal transition. Front. Cell Dev Biol..

[11] Chen C, Tang Y, Zhu X, Yang J, Liu Z, Chen Y (2023). P311 promotes IL-4 receptor‒mediated M2 polarization of macrophages to enhance angiogenesis for efficient skin wound healing. J Investig Dermatol..

[12] Pan D, Zhe X, Jakkaraju S, Taylor GA, Schuger L (2002). P311 induces a TGF-beta1-independent, nonfibrogenic myofibroblast phenotype. J Clin Invest.

[13] Tan J, Peng X, Luo G, Ma B, Cao C, He W (2010). Investigating the role of P311 in the hypertrophic scar. PLoS One.

[14] Duan FF, Barron G, Meliton A, Mutlu GM, Dulin NO, Schuger L (2019). P311 promotes lung fibrosis via stimulation of transforming growth factor-beta1, -beta2, and -beta3 translation. Am J Respir Cell Mol Biol..

[15] Guimaraes EL, Stradiot L, Mannaerts I, Schroyen B, van Grunsven LA (2015). P311 modulates hepatic stellate cells migration. Liver Int.

[16] Li Q, Fu L, Wu D, Wang J (2023). NREP is a diagnostic and prognostic biomarker, and promotes gastric cancer cell proliferation and angiogenesis. Biochem Genet..

[17] Mariani L, McDonough WS, Hoelzinger DB, Beaudry C, Kaczmarek E, Coons SW (2001). Identification and validation of P311 as a glioblastoma invasion gene using laser capture microdissection. Cancer Res..

[18] Liu Y, Zhou X, Hu N, Wang C, Zhao L (2020). P311 regulates distal lung development via its interaction with several binding proteins. Mech Dev..

[19] Lunt SY, Vander Heiden MG (2011). Aerobic glycolysis: meeting the metabolic requirements of cell proliferation. Annu Rev Cell Dev Biol..

[20] Orang AV, Petersen J, McKinnon RA, Michael MZ (2019). Micromanaging aerobic respiration and glycolysis in cancer cells. Mol Metab..

[21] Jiang Y, Zhang M, Yu D, Hou G, Wu J, Li F (2022). CircRBM33 downregulation inhibits hypoxia-induced glycolysis and promotes apoptosis of breast cancer cells via a microRNA-542-3p/HIF-1α axis. Cell Death Discov..

[22] Yue MM, Lv K, Meredith SC, Martindale JL, Gorospe M, Schuger L (2014). Novel RNA-binding protein P311 binds eukaryotic translation initiation factor 3 subunit b (eIF3b) to promote translation of transforming growth factor beta1-3 (TGF-beta1-3). J Biol Chem..

[23] Rodriguez-Garcia A, Samso P, Fontova P, Simon-Molas H, Manzano A, Castano E (2017). TGF-beta1 targets Smad, p38 MAPK, and PI3K/Akt signaling pathways to induce PFKFB3 gene expression and glycolysis in glioblastoma cells. FEBS J.

[24] Smith ER, Hewitson TD (2020). TGF-beta1 is a regulator of the pyruvate dehydrogenase complex in fibroblasts. Sci. Rep..

[25] Yao Z, Li H, He W, Yang S, Zhang X, Zhan R (2017). P311 accelerates skin wound reepithelialization by promoting epidermal stem cell migration through RhoA and Rac1 activation. Stem Cells Dev..

[26] McDonough WS, Tran NL, Berens ME (2005). Regulation of glioma cell migration by serine-phosphorylated P311. Neoplasia.

[27] Shi J, Badri KR, Choudhury R, Schuger L (2006). P311-induced myofibroblasts exhibit ameboid-like migration through RalA activation. Exp Cell Res.

[28] Li Q, Qin T, Bi Z, Hong H, Ding L, Chen J (2020). Rac1 activates non-oxidative pentose phosphate pathway to induce chemoresistance of breast cancer. Nat Commun.

[29] Vaupel P, Mayer A, Höckel M (2004). Tumor hypoxia and malignant progression. Methods Enzymol..

[30] Xiang L, Semenza GL (2019). Hypoxia-inducible factors promote breast cancer stem cell specification and maintenance in response to hypoxia or cytotoxic chemotherapy. Adv Cancer Res.

[31] Liu X, Xie P, Hao N, Zhang M, Liu Y, Liu P et al. HIF-1-regulated expression of calreticulin promotes breast tumorigenesis and progression through Wnt/β-catenin pathway activation. Proc Natl Acad Sci USA 118 (2021). 10.1073/pnas.2109144118 10.1073/pnas.2109144118 PMC8612225 34706936

[32] Graeber TG, Osmanian C, Jacks T, Housman DE, Koch CJ, Lowe SW (1996). Hypoxia-mediated selection of cells with diminished apoptotic potential in solid tumours. Nature.

[33] Talks KL, Turley H, Gatter KC, Maxwell PH, Pugh CW, Ratcliffe PJ (2000). The expression and distribution of the hypoxia-inducible factors HIF-1alpha and HIF-2alpha in normal human tissues, cancers, and tumor-associated macrophages. Am J Pathol..

[34] Vaupel P, Mayer A (2007). Hypoxia in cancer: significance and impact on clinical outcome. Cancer Metastasis Rev..

[35] Semenza GL, Nejfelt MK, Chi SM, Antonarakis SE (1991). Hypoxia-inducible nuclear factors bind to an enhancer element located 3’ to the human erythropoietin gene. Proc Natl Acad Sci USA.

[36] Wang GL, Semenza GL (1995). Purification and characterization of hypoxia-inducible factor 1. J Biol Chem..

[37] Wang R, Godet I, Yang Y, Salman S, Lu H, Lyu Y et al. Hypoxia-inducible factor-dependent ADAM12 expression mediates breast cancer invasion and metastasis. Proc Natl Acad Sci USA. 2021;118:e2020490118. 10.1073/pnas.2020490118 10.1073/pnas.2020490118 PMC8126789 33952697

[38] Shigeta K, Hasegawa M, Hishiki T, Naito Y, Baba Y, Mikami S (2023). IDH2 stabilizes HIF-1α-induced metabolic reprogramming and promotes chemoresistance in urothelial cancer. EMBO J..

[39] Sharma D, Singh M, Rani R (2022). Role of LDH in tumor glycolysis: regulation of LDHA by small molecules for cancer therapeutics. Semin Cancer Biol..

[40] Lu B, Nie XH, Yin R, Ding P, Su ZZ, Qiu S (2023). PGAM4 silencing inhibited glycolysis and chemoresistance to temozolomide in glioma cells. Cell Biol Int.

[41] Sahuri-Arisoylu M, Mould RR, Shinjyo N, Bligh SWA, Nunn AVW, Guy GW (2021). Acetate induces growth arrest in colon cancer cells through modulation of mitochondrial function. Front Nutr.

[42] Kim D, Khin PP, Lim OK, Jun HS (2022). LPA/LPAR1 signaling induces PGAM1 expression via AKT/mTOR/HIF-1α pathway and increases aerobic glycolysis, contributing to keratinocyte proliferation. Life Sci..

[43] Qiu Z, Wang C, Huang P, Yuan Y, Shi Y, Lin Z (2023). RFX6 facilitates aerobic glycolysis-mediated growth and metastasis of hepatocellular carcinoma through targeting PGAM1. Clin Transl Med..

[44] Gao L, Yang F, Tang D, Xu Z, Tang Y, Yang D (2023). Mediation of PKM2-dependent glycolytic and non-glycolytic pathways by ENO2 in head and neck cancer development. J Exp Clin Cancer Res.

[45] Lin J, Xia L, Oyang L, Liang J, Tan S, Wu N (2022). The POU2F1-ALDOA axis promotes the proliferation and chemoresistance of colon cancer cells by enhancing glycolysis and the pentose phosphate pathway activity. Oncogene.

[46] Mazarei M, Lennon KA, Puthoff DP, Rodermel SR, Baum TJ (2003). Expression of an Arabidopsis phosphoglycerate mutase homologue is localized to apical meristems, regulated by hormones, and induced by sedentary plant-parasitic nematodes. Plant Mol Biol.

[47] Zhou R, Ni W, Qin C, Zhou Y, Li Y, Huo J (2022). A functional loop between YTH domain family protein YTHDF3 mediated m(6)A modification and phosphofructokinase PFKL in glycolysis of hepatocellular carcinoma. J Exp Clin Cancer Res.

[48] Massari F, Ciccarese C, Santoni M, Iacovelli R, Mazzucchelli R, Piva F (2016). Metabolic phenotype of bladder cancer. Cancer Treat Rev..

[49] Jiang T, Liang YS, Gu Y, Yao FC, Liu YF, Zhang KX (2023). Different reoxygenation rates induce different metabolic, apoptotic and immune responses in Golden Pompano (Trachinotus blochii) after hypoxic stress. Fish Shellfish Immunol..

[50] Xu H, Li YF, Yi XY, Zheng XN, Yang Y, Wang Y (2023). ADP-dependent glucokinase controls metabolic fitness in prostate cancer progression. Mil Med Res.

[51] Qian X, Li X, Shi Z, Xia Y, Cai Q, Xu D (2019). PTEN suppresses glycolysis by dephosphorylating and inhibiting autophosphorylated PGK1. Mol Cell.

[52] Yang S, Wu H, Li Y, Li L, Xiang J, Kang L (2023). Inhibition of PFKP in renal tubular epithelial cell restrains TGF-β induced glycolysis and renal fibrosis. Cell Death Dis.

[53] Jin X, Wang D, Lei M, Guo Y, Cui Y, Chen F (2022). TPI1 activates the PI3K/AKT/mTOR signaling pathway to induce breast cancer progression by stabilizing CDCA5. J Transl Med..

[54] Liu BHM, Tey SK, Mao X, Ma APY, Yeung CLS, Wong SWK (2021). TPI1-reduced extracellular vesicles mediated by Rab20 downregulation promotes aerobic glycolysis to drive hepatocarcinogenesis. J Extracell Vesicles.

[55] Yu WS, Jeong SJ, Kim JH, Lee HJ, Song HS, Kim MS (2011). The genome-wide expression profile of 1,2,3,4,6-penta-O-galloyl-β-D-glucose-treated MDA-MB-231 breast cancer cells: molecular target on cancer metabolism. Mol Cells.

[56] Li J, Shi X, Chen Z, Xu J, Zhao R, Liu Y (2023). Aldehyde dehydrogenase 2 alleviates mitochondrial dysfunction by promoting PGC-1α-mediated biogenesis in acute kidney injury. Cell Death Dis..

[57] Wang Z, Dong C (2019). Gluconeogenesis in cancer: function and regulation of PEPCK, FBPase, and G6Pase. Trends Cancer.

[58] Huangyang P, Li F, Lee P, Nissim I, Weljie AM, Mancuso A (2020). Fructose-1,6-bisphosphatase 2 inhibits sarcoma progression by restraining mitochondrial biogenesis. Cell Metab..

[59] Li H, Wang J, Xu H, Xing R, Pan Y, Li W (2013). Decreased fructose-1,6-bisphosphatase-2 expression promotes glycolysis and growth in gastric cancer cells. Mol. Cancer.

[60] Zhang L, Liu Z, Dong Y, Kong L (2021). Epigenetic targeting of SLC30A3 by HDAC1 is related to the malignant phenotype of glioblastoma. IUBMB Life.

[61] Zhang H, Xie T, Shui Y, Qi Y (2020). Knockdown of PLCB2 expression reduces melanoma cell viability and promotes melanoma cell apoptosis by altering Ras/Raf/MAPK signals. Mol Med Rep.

[62] Wang D, Tang F, Liu X, Fan Y, Zheng Y, Zhuang H (2021). Expression and tumor-promoting effect of tyrosine phosphatase receptor type N (PTPRN) in human glioma. Front Oncol..

[63] Song X, Jiao X, Yan H, Yu L, Jiang L, Zhang M (2021). Overexpression of PTPRN promotes metastasis of lung adenocarcinoma and suppresses NK cell cytotoxicity. Front Cell Dev Biol..

[64] Niu G, Deng L, Zhang X, Hu Z, Han S, Xu K (2020). GABRD promotes progression and predicts poor prognosis in colorectal cancer. Open Med.

[65] Yuan Y, Ping W, Zhang R, Hao Z, Zhang N (2022). DEPDC1B collaborates with GABRD to regulate ESCC progression. Cancer Cell Int.

[66] Li P, Weng Z, Li P, Hu F, Zhang Y, Guo Z (2021). BATF3 promotes malignant phenotype of colorectal cancer through the S1PR1/p-STAT3/miR-155-3p/WDR82 axis. Cancer Gene Ther..

[67] Bertagnolo V, Benedusi M, Querzoli P, Pedriali M, Magri E, Brugnoli F (2006). PLC-beta2 is highly expressed in breast cancer and is associated with a poor outcome: a study on tissue microarrays. Int J Oncol.

