# Supplementary material for: NREP, transcriptionally upregulated by HIF-1α, aggravates breast cancer cell growth and metastasis by promoting glycolysis
Source: Cell Death Discov. 2024 May 2;10:210. doi: 10.1038/s41420-024-01951-2 (PMC11066005; doi:10.1038/s41420-024-01951-2)
Supplement: Supplementary file 1 — Supplementary data-Revised [file 41420_2024_1951_MOESM1_ESM.docx]

Table S1 Primer sequences that used for qPCR.

| ADGRE1 Forward (F) | TGTGCCTTTGGTTATGG |
| --- | --- |
| ADGRE1 Reverse  (R) | TTTAGCGTTGAGACTTCG |
| BATF3 F | TGACAGGAAGGTCCGAAGG |
| BATF3 R | GCACAAAGTTCATAGGGCAGA |
| GABRD F | CCATTGTCCTCTTCTCCCTCT |
| GABRD R | CCCTCCTTCTTCGTCTCCC |
| NTNG2 F | CTGCGAATGCTACGGTCAC |
| NTNG2 R | CACGGAGCCTATCTGGTTG |
| PLCB2 F | CTGGAACAGATACGGGAGAT |
| PLCB2 R | GCCTTTGCTATGAGTGGGT |
| PNMT F | ACGGTGGTGCCAGTGTCT |
| PNMT R | AGCCCAGGCGAAGAAGA |
| PTPRN F | AGCATCGGCTTCCACAAC |
| PTPRN R | CAGCAAGGCAGGTTCGTAA |
| SLC30A3 F | GCCCTTCCACCACTGCCACA |
| SLC30A3 R | AGATACCCGCCGACCACCTC |
| SMIM10L2B F | CTTGGCGGTTCGGCTGTC |
| SMIM10L2B R | CTTGCAGGCGGACGTTGA |
| SNCB F | GGAAGCAAGACCCGAGAA |
| SNCB R | CTGTGGCTGCTGCGATG |
| NREP F | TTATTACCCAGAACTCTTTGTC |
| NREP R | CAGCGTTTGTCTCATCGT |
| HIF-1α F | AGTGTACCCTAACTAGCCG |
| HIF-1α R | CACAAATCAGCACCAAGC |
| CCNA2 F | TTAGGGAAATGGAGGTTA |
| CCNA2 R | TAGTTCACAGCCAAATGC |
| CCNE1 F | GGATGTTGACTGCCTTGA |
| CCNE1 R | CACCACTGATACCCTGAAA |
| CD31 F | AAGATAGCCTCAAAGTCG |
| CD31 R | CTGGGCATCATAAGAAAT |
| Ki67 F | AGATGTGCTCTGGGTTAC |
| Ki67 R | TGATGGTTGAGGCTGT |
| E-cadherin F | CTGAGAACGAGGCTAACG |
| E-cadherin R | GTCCACCATCATCATTCAATAT |
| N-cadherin F | TCCTGCTTATCCTTGTGCTG |
| N-cadherin R | TCCTGGTCTTCTTCTCCTCC |


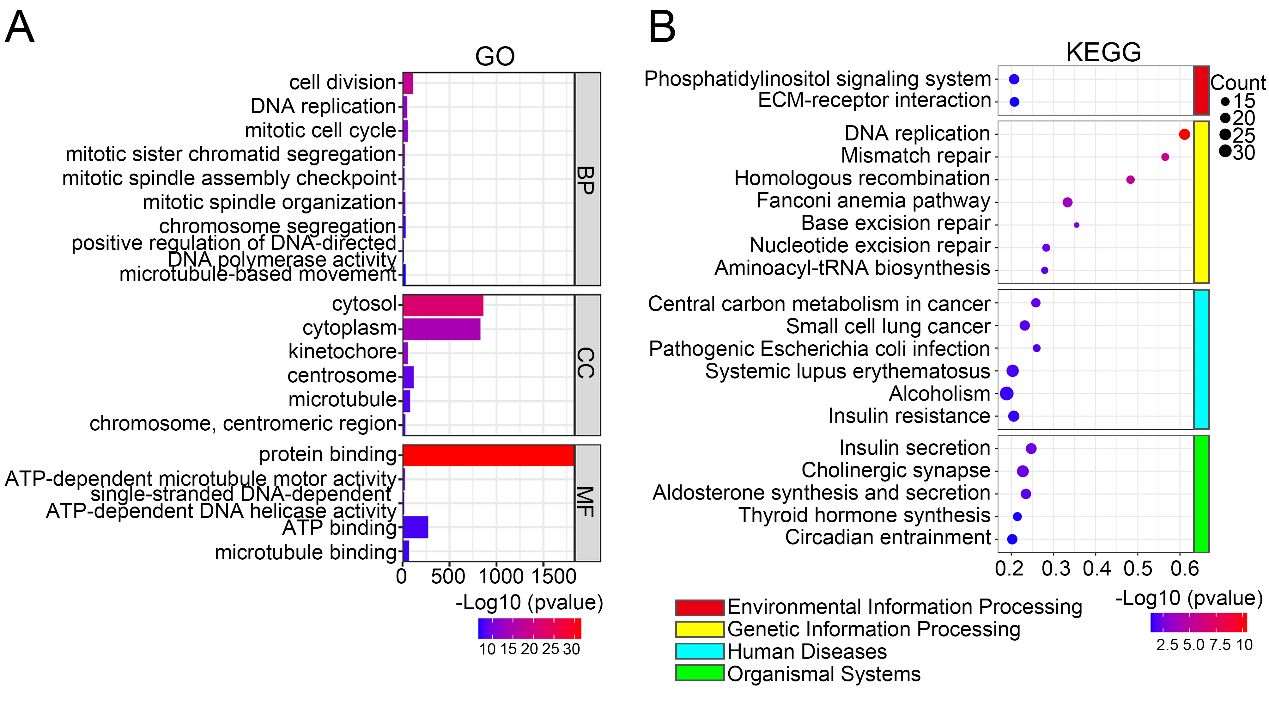


Figure S1 The top 20 enriched GO terms (A) and all significantly enriched KEGG pathways (B) of DEGs from RNA-sequencing results.


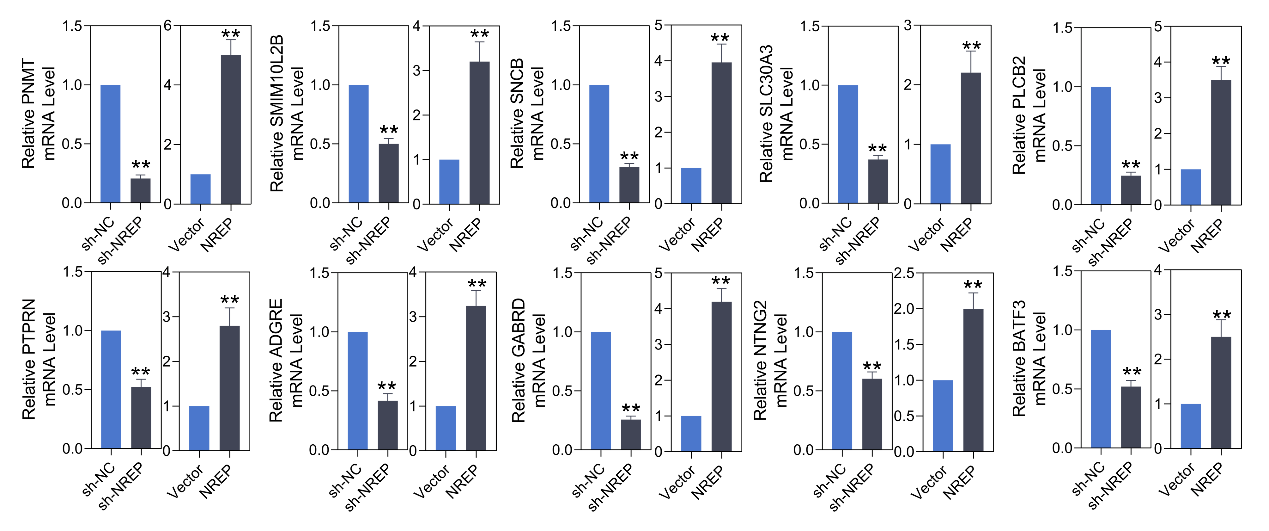


Figure S2 The mRNA expression of the top 10 downregulated DEGs in NREP silenced or upregulated MDA-MB-468 cells.
