# Supplementary material for: NREP, transcriptionally upregulated by HIF-1α, aggravates breast cancer cell growth and metastasis by promoting glycolysis
Source: Cell Death Discov. 2024 May 2;10:210. doi: 10.1038/s41420-024-01951-2 (PMC11066005; doi:10.1038/s41420-024-01951-2)
Supplement: Supplementary file 2 — Supplementary material-Western Blot [file 41420_2024_1951_MOESM2_ESM.pdf]

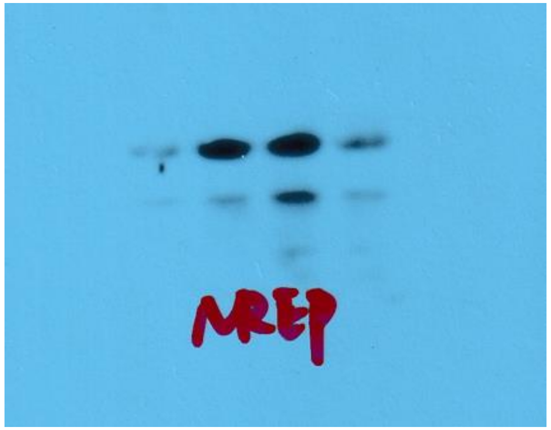
NREP

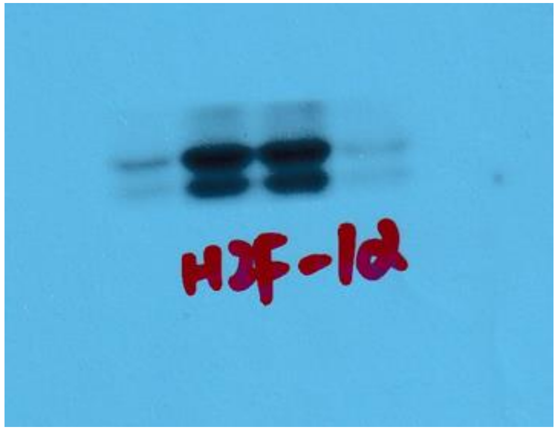
HIF-1α

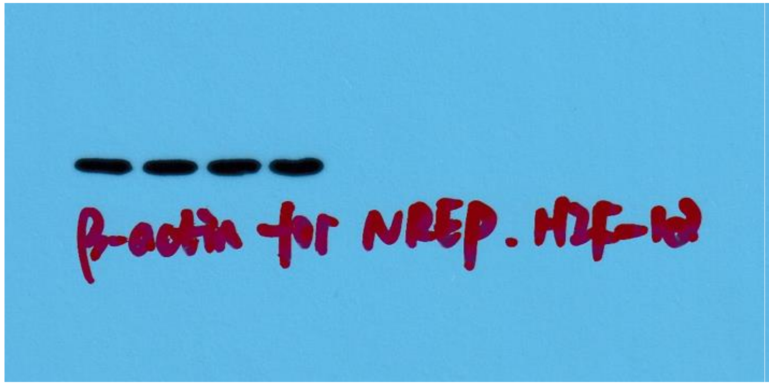
β-actin for NREP.

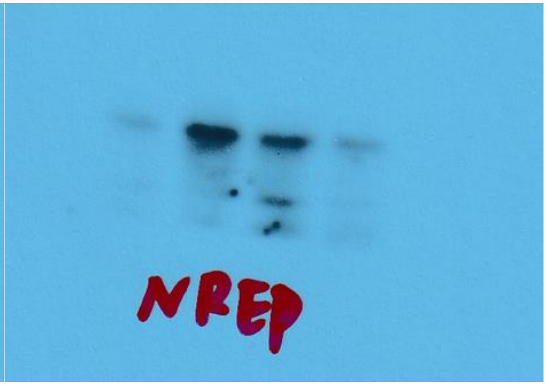
NREP

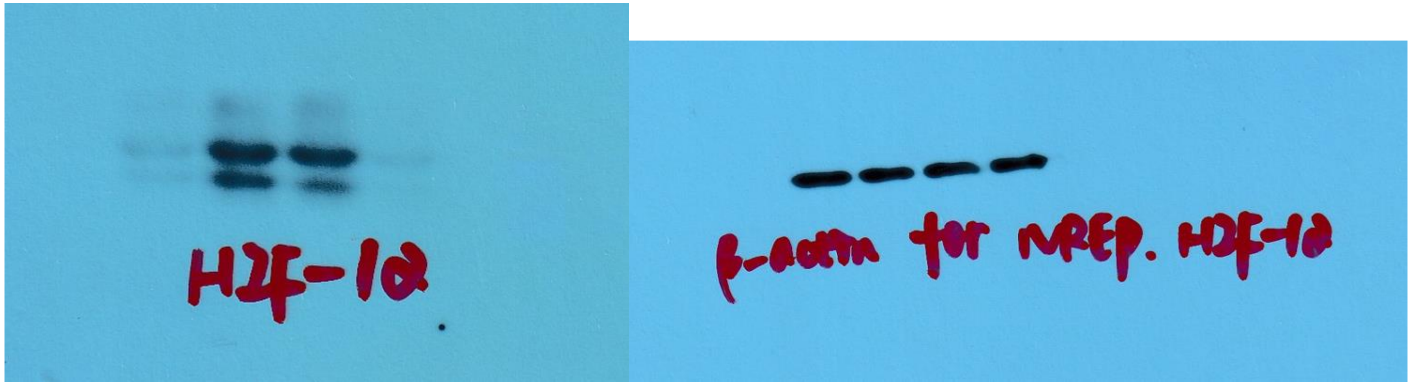
HIF-1α
β-actin for NREP. HIF-1α

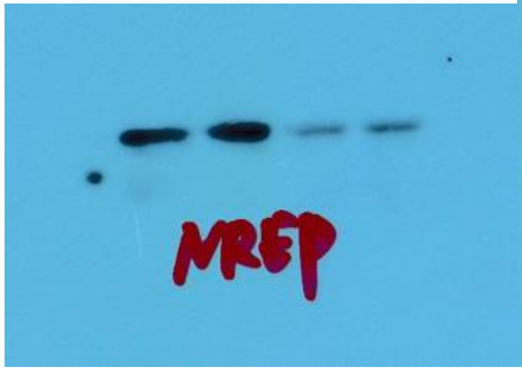
NREP

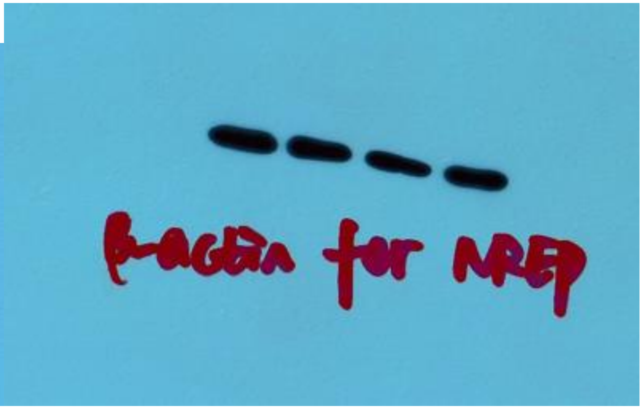
β-actin for NREP

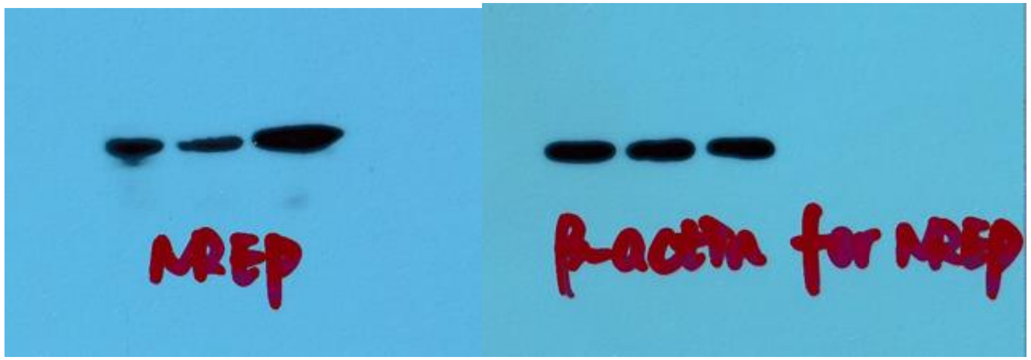
NREP
β-actin for NREP

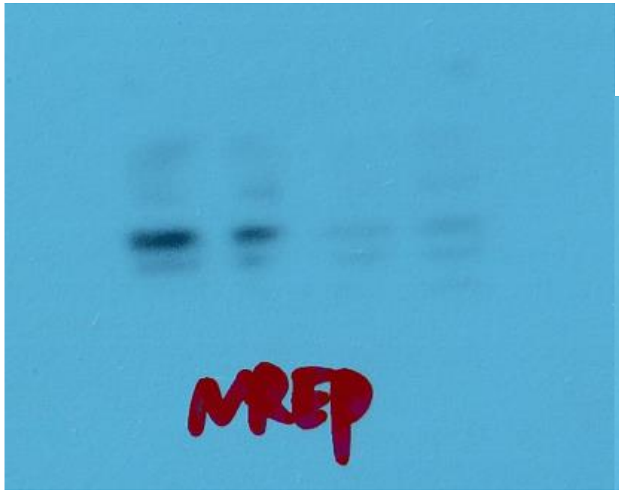
NREP

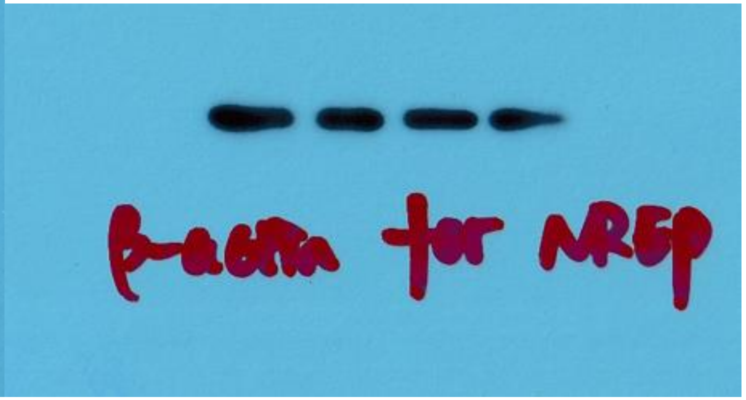
β-actin for NREP

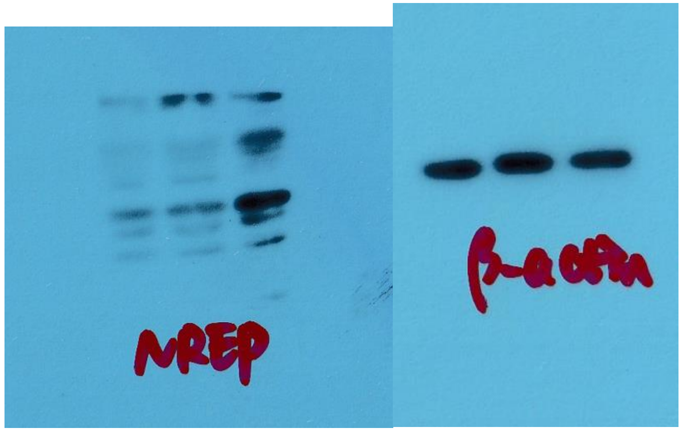
NREP
β-actin

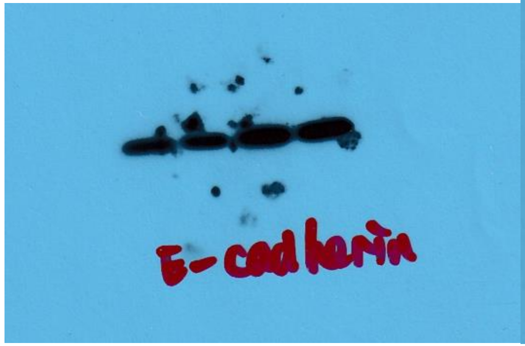
E-cadherin

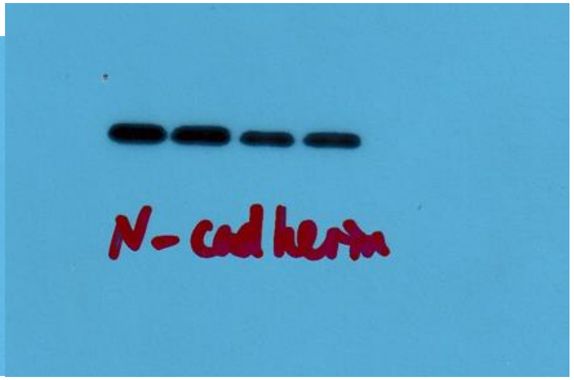
N-cadherin

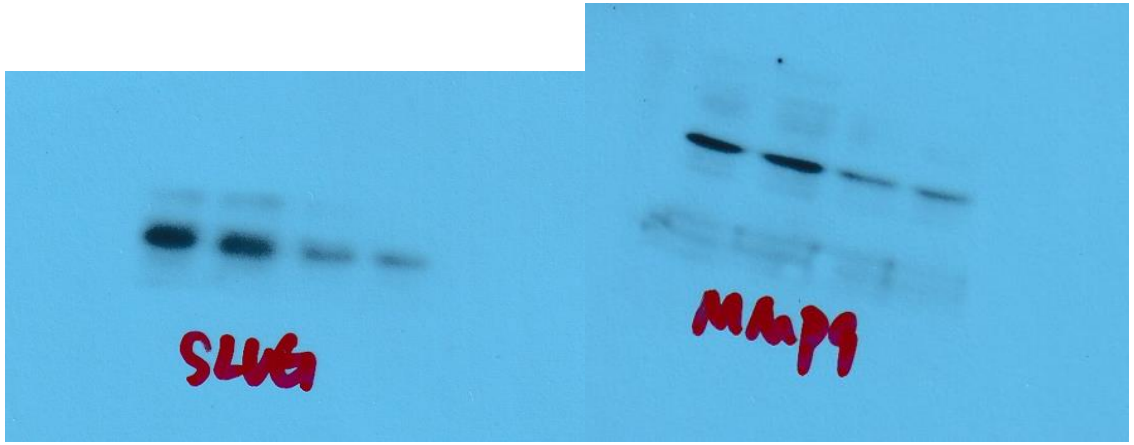
SLUG
MMP9

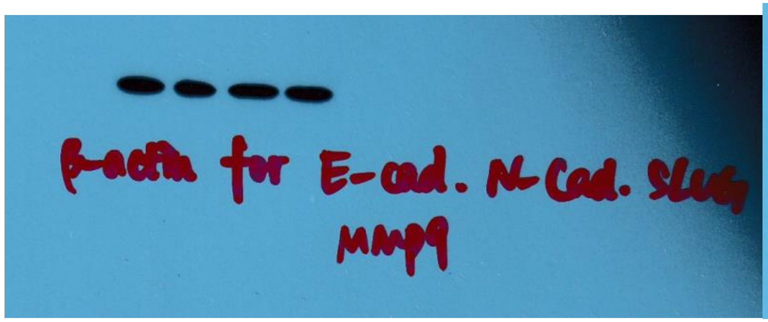
β-actin for E-cad. N-Cad. SLUG
MMP9

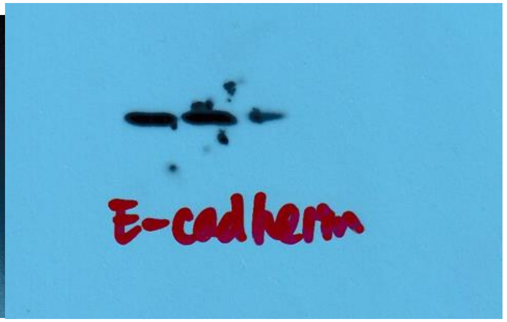
E-cadherin

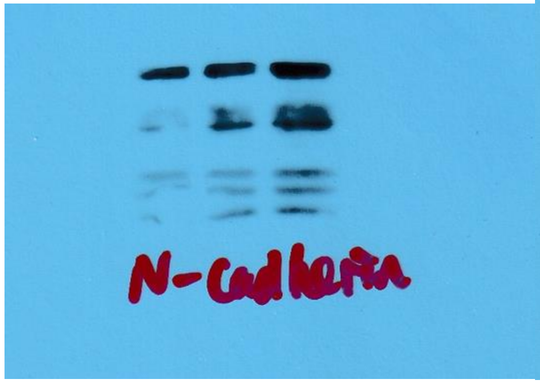
N-cadherin
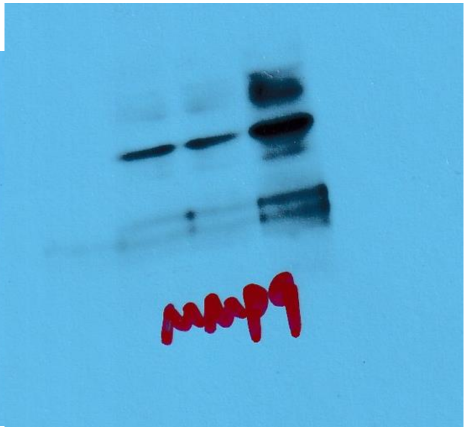
MMP9

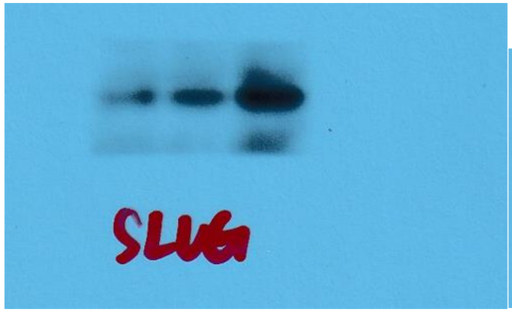
SLUG

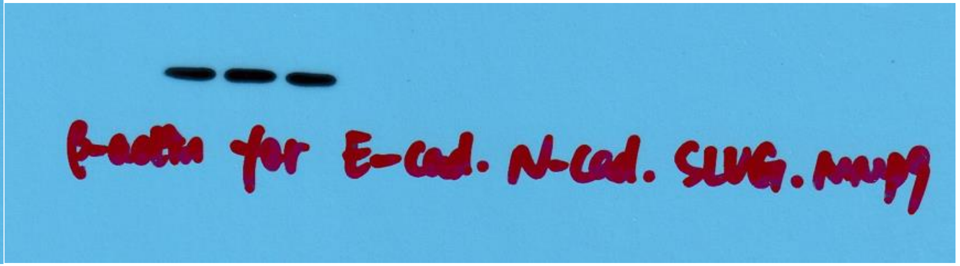
β-actin for E-cad. N-cad. SLUG. MMP9

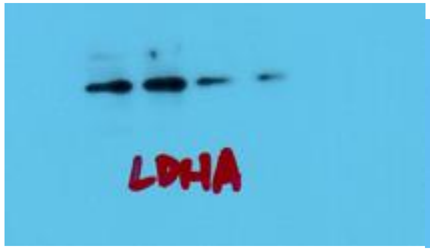
LDHA

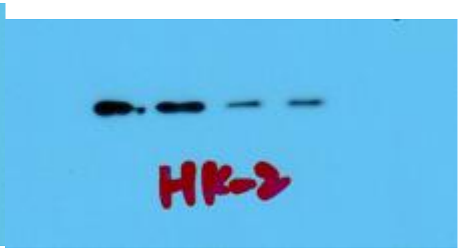
HK-2

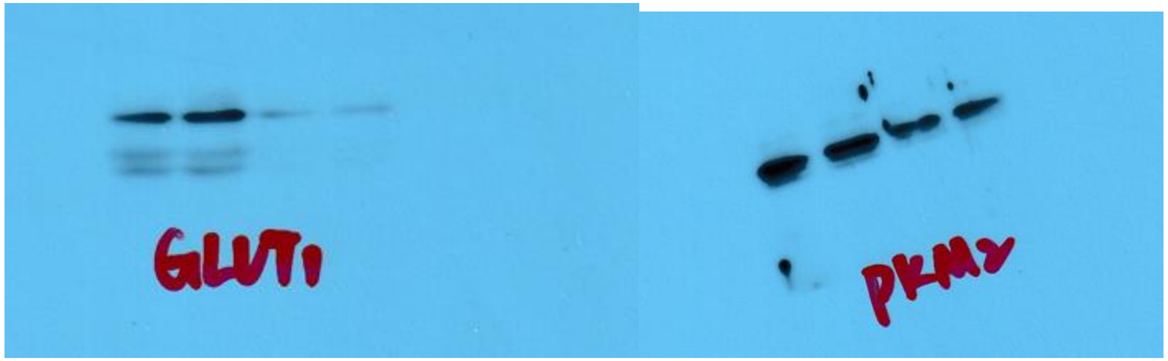
GLUT1
PKM2

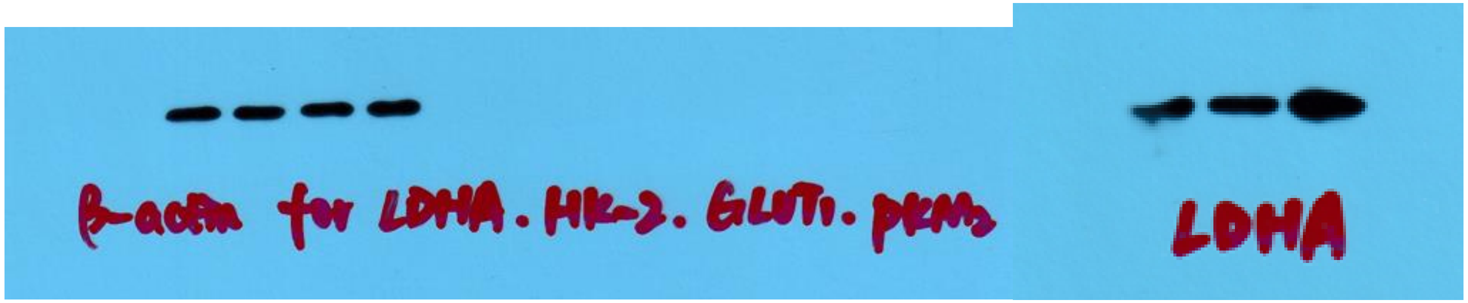
β-actin for LDHA. HK-2. GLUT1. pKM2
LDHA

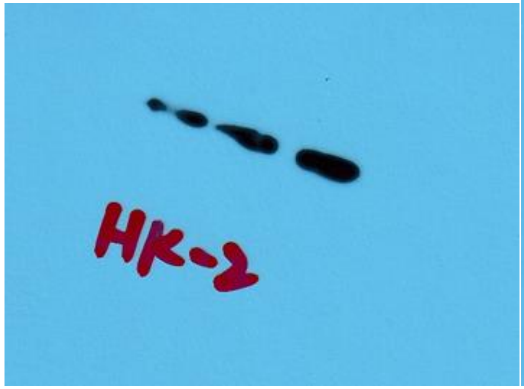
HK-2

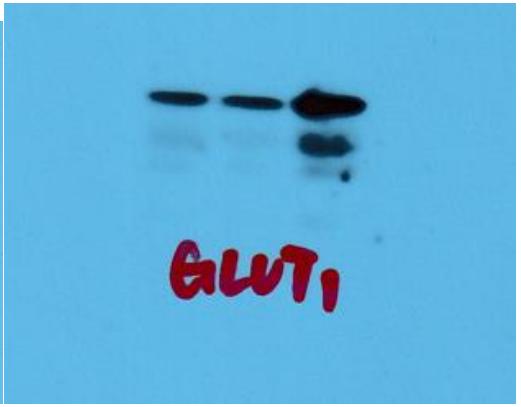
GLUT1

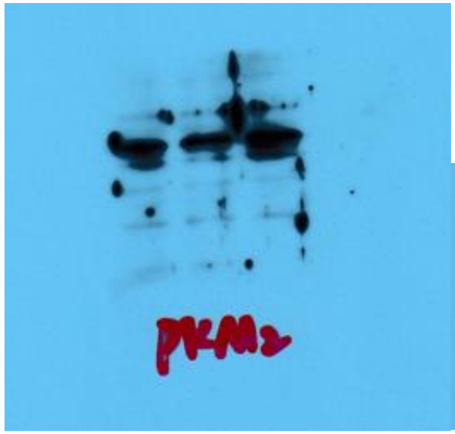
pKM2

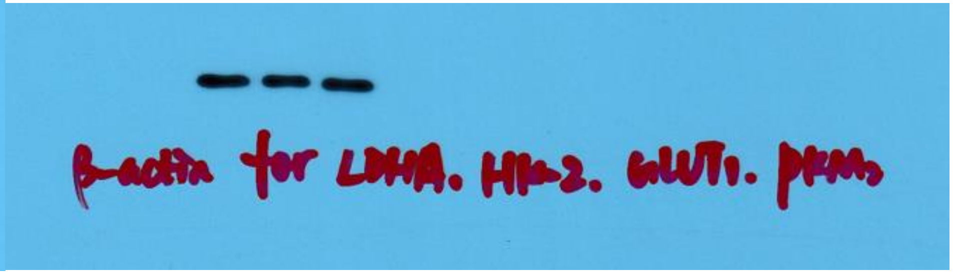
β-actin for LDHA. HK-2. GLUT1. pKM2

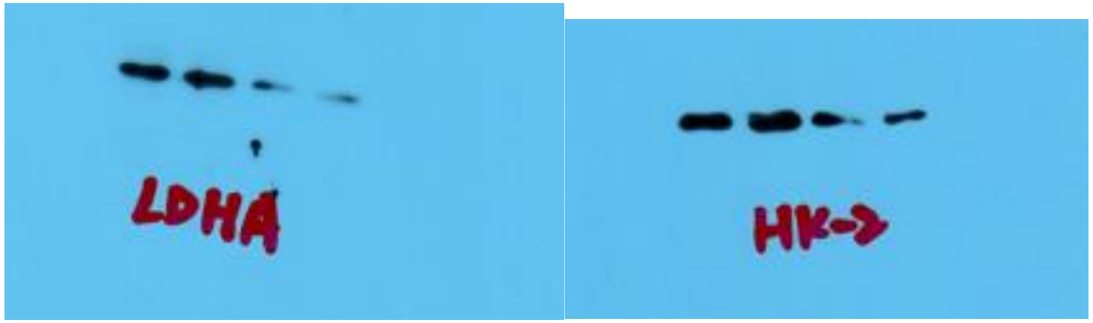
LDHA
HK-2

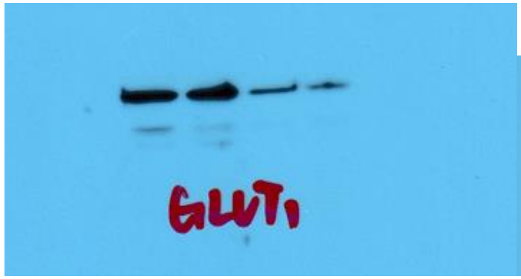
GLUT1

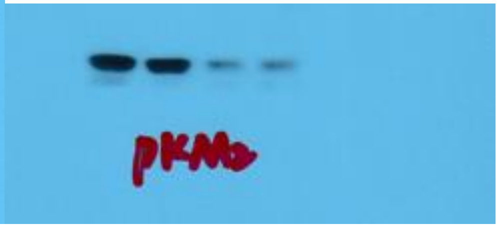
pKM2

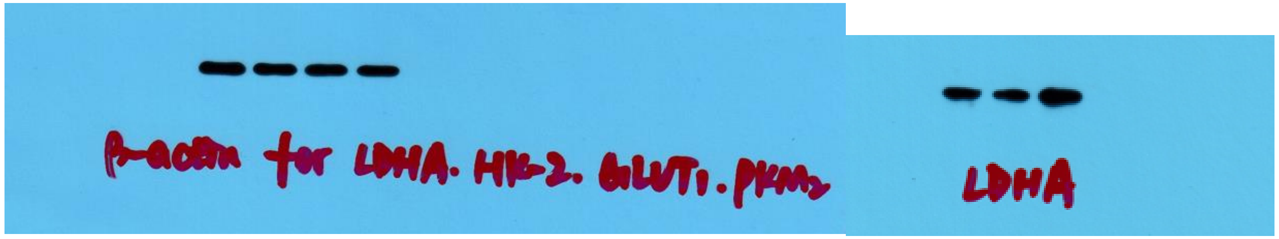
β-actin for LDHA. HK-2. GLUT1. PKM2
LDHA

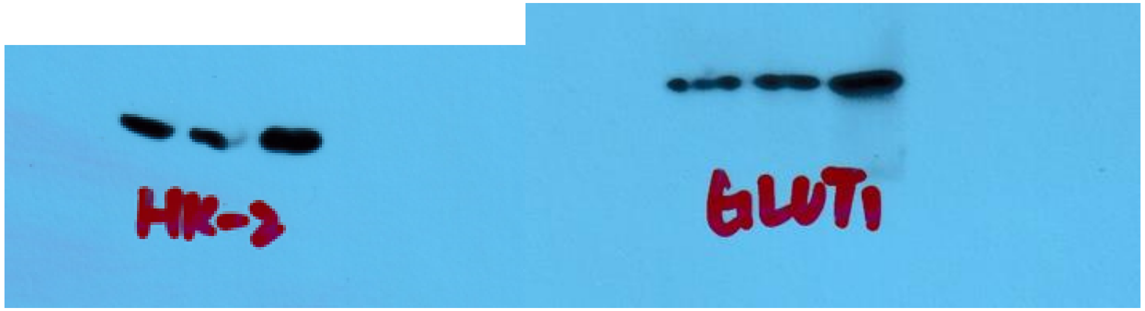

HK-2
GLUT1

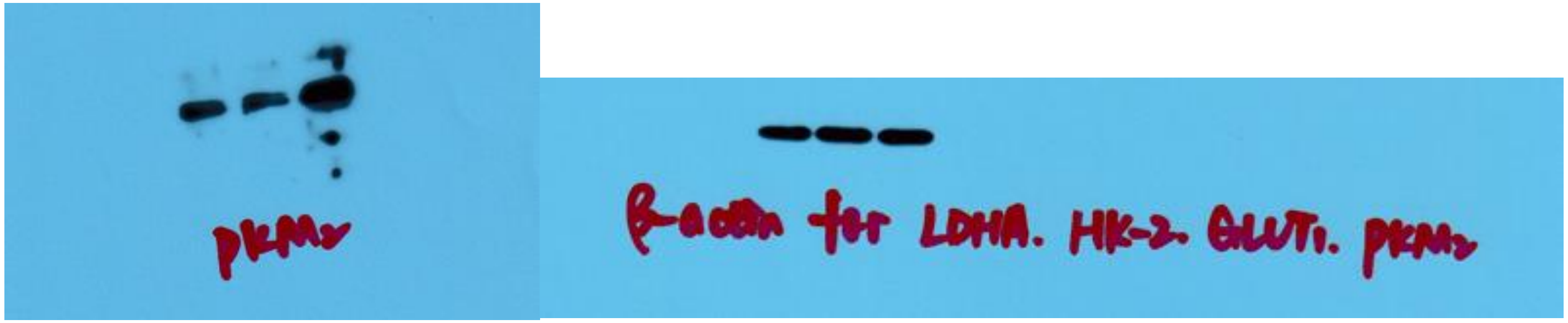
pKM2
β-actin for LDHA. HK-2. GLUT1. pKM2
